# Diagnostic performance of dual-energy computed tomography in detecting anterior cruciate ligament injuries: a systematic review and meta-analysis

**DOI:** 10.1007/s00256-024-04833-x

**Published:** 2024-11-21

**Authors:** Parya Valizadeh, Payam Jannatdoust, Mohammad-Taha Pahlevan-Fallahy, Sara Bagherieh, Paniz Adli, Melika Amoukhteh, Amir Hassankhani, George R. Matcuk, Ali Gholamrezanezhad

**Affiliations:** 1 School of Medicine, Tehran University of Medical Sciences, Tehran, Iran; 2 School of Medicine, Isfahan University of Medical Sciences, Isfahan, Iran; 3 College of Letters and Science, University of California, Berkeley, CA USA; 4 Department of Radiology, Keck School of Medicine, University of Southern California (USC), 1441 Eastlake Ave Ste 2315, Los Angeles, CA 90089 USA; 5 Department of Radiology, Mayo Clinic, Rochester, MN USA; 6 Department of Radiology, Cedars Sinai Medical Center, Los Angeles, CA USA; 7 Department of Radiology, Los Angeles General Hospital, Los Angeles, CA USA

**Keywords:** Anterior cruciate ligament, Ligament injury, Dual-energy computed tomography, Knee injury, Meta-analysis

## Abstract

**Objective:**

Anterior cruciate ligament (ACL) injuries are common and lead to significant physical limitations. While MRI is the diagnostic gold standard, its use is restricted in acute trauma cases due to contraindications and longer imaging times. Dual-energy computed tomography (DECT) has emerged as a potential alternative. This meta-analysis evaluates the diagnostic accuracy of DECT for ACL injuries.

**Materials and methods:**

Following PRISMA guidelines, a comprehensive literature search was conducted using PubMed, Web of Science, Scopus, and Embase for studies published up to June 2024. Studies that provided diagnostic accuracy data for DECT in ACL ruptures were included. Metrics of diagnostic accuracy were aggregated using a bivariate random effects model.

**Results:**

The meta-analysis, which included five studies with a total of 191 patients, found that DECT had a pooled sensitivity of 88.1% (95% CI, 78.0–93.9%) and a specificity of 82.0% (95% CI, 62.0–92.7%) for diagnosing ACL ruptures, with an AUC of 0.92 (95% CI, 0.72–0.96). For complete ruptures, sensitivity was 83.2% (95% CI, 68.2–92.0%), and specificity was 94.9% (95% CI, 92.2–96.7%), with an AUC of 0.96 (95% CI, 0.81–0.98). In acute/subacute settings, sensitivity was 89.4% (95% CI, 76.8–95.6%), and specificity was 82.1% (95% CI, 56.2–94.2%), with an AUC of 0.93 (95% CI, 0.71–0.97).

**Conclusion:**

Our findings suggest that DECT is a valuable diagnostic tool for ACL injuries, particularly as an adjunct or alternative when MRI is unavailable or contraindicated, enabling timely and accurate diagnosis.

**Supplementary Information:**

The online version contains supplementary material available at 10.1007/s00256-024-04833-x.

## Introduction

The anterior cruciate ligament (ACL) is a crucial stabilizer of the knee joint. Injuries to the ACL can cause severe pain, swelling, limited range of motion, and an inability to participate in certain sports, with some patients experiencing restricted physical capacity even after the initial symptoms subside [1–3]. The ACL is also the most frequently injured ligament in the knee joint, with an incidence rate of 0.08 per 1000 exposures in female athletes and 0.05 per 1000 exposures in male athletes [3]. ACL injuries are classified into three grades: 1 = mild stretching, 2 = partial tear with knee instability, and 3 = complete tear with severe pain and functional loss. Treatment depends on the severity, ranging from rehabilitation to surgical intervention. Timely diagnosis is critical, as delayed or neglected injuries can lead to chronic knee pain, joint instability, and knee osteoarthritis later on [4].

Although the diagnosis of an ACL injury can be ventured clinically, considering the trauma mechanism and medical examination findings (e.g., Lachman and anterior drawer tests), imaging is required to confirm the diagnosis, evaluate for other associated injuries, and for treatment planning. Among all the available imaging modalities that can be utilized to examine internal derangement, magnetic resonance imaging (MRI) has been proven to be the most accurate and currently serves as the imaging reference standard of soft tissue examination [2]. However, the use of MRI may be limited by contraindications such as the presence of metallic implants, pacemakers, or claustrophobia. MRI may also not be readily available in the acute traumatic setting, with many patients seen in urgent care or emergency departments needing to return for subsequent outpatient imaging, with increasingly long backlogs and wait times.

As a result, alternative imaging modalities have been proposed for diagnosing ACL injuries, with dual-energy computed tomography (DECT) standing out. DECT is an imaging technique that enables detailed investigation of tissues with different attenuation properties [5]. Increasingly, CT scanners with this technology are being installed and available for use in the emergency setting. Previous studies have shown that DECT has acceptable diagnostic accuracy for diagnosing ACL injuries compared to the reference standard. For example, Peltola et al. [2] and Liu et al. [6] reported accuracies of 93% and 97.5%, respectively.

In this systematic review and meta-analysis, we aim to comprehensively assess all existing literature on the use of DECT for diagnosing ACL injuries and to meta-analyze its diagnostic accuracy metrics.

## Methods

This systematic review adheres to the Preferred Reporting Items for Systematic Reviews and Meta-Analyses (PRISMA) guidelines [7]. The literature search began on June 8, 2024, and encompassed four major databases: PubMed, Web of Science, Scopus, and Embase. The search terms included keywords related to dual-energy computed tomography and cruciate ligaments. Additionally, the references of the selected studies were manually reviewed to ensure comprehensive coverage. Two co-authors independently evaluated each article’s title, abstract, and full text, with any disagreements resolved through consultation with a senior co-author. Deduplication and screening were conducted using the Rayyan platform. The complete search strategy is available in Supplementary Table 1.

Studies assessing the diagnostic accuracy of DECT for ACL ruptures were included if they provided sufficient data to calculate true positive (TP), true negative (TN), false positive (FP), and false negative (FN) results. No restrictions were placed on publication date, study design, country of origin, reference test used, or patient characteristics. However, non-English studies, case series, case reports, editorial comments, conference abstracts, and review articles were excluded.

For each included study, the following details were extracted: first author’s name, publication year, study design, sample size, participant characteristics, type of ACL rupture (complete or partial), injury stage (acute, subacute, or chronic), reference test, time intervals from injury to DECT/reference test, interpreter, device type, radiation dose, and diagnostic accuracy metrics for DECT.

The quality assessment was performed using the QUADAS-2 (Diagnostic Accuracy Studies-2) tool to evaluate the quality of the included studies [8]. Independent assessments were conducted for potential bias and concerns about applicability in the four main domains of the QUADAS-2 tool: patient selection, index test, reference standard, and flow and timing. The evaluations for each domain were based on specific criteria mentioned in the tool. Each domain was rated as “low,” “high,” or “unclear.”

Generative AI tools, specifically ChatGPT (OpenAI, GPT-4 architecture), were used to assist in language correction, improving the clarity and readability of the manuscript. No generative AI tools were used for data analysis, research design, interpretation of results, or the formation of scientific conclusions. The authors carefully reviewed, revised, and approved all AI-generated edits to ensure accuracy and integrity.

## Statistical analysis

A random effects diagnostic test accuracy (DTA) model was used to aggregate the diagnostic accuracy metrics across studies, implementing the bivariate model developed by Reitsma et al. [9]. Summary receiver operating characteristic (SROC) curves were created from the bivariate meta-analysis data, incorporating study-specific estimates weighted in the random effects univariate diagnostic odds ratio (DOR) model. To calculate the AUC and its confidence interval (CI), 2000 sample bootstrapping was performed using the bivariate model [10].

We hypothesized that diagnostic accuracy might vary based on rupture type (complete vs. partial), injury stage (acute/subacute vs. chronic), and the reference standard used (MRI vs. arthroscopy). Subgroup analyses were conducted to explore potential variations in diagnostic performance based on these factors.

Furthermore, heterogeneity was assessed using the I2 metric according to Holling et al.’s method, where an I2 confidence interval over 50% indicated significant heterogeneity [11]. Sensitivity analyses employing the univariate meta-analysis of the DOR were conducted for each subgroup analysis to detect potential outliers when significant heterogeneity was present. If any outliers were found, a re-analysis after the exclusion of the outlier study was conducted to confirm the results.

Likelihood ratio scattergrams and Fagan plot studies were used to evaluate the clinical relevance of the diagnostic accuracy findings. Positive likelihood ratios greater than ten were considered suitable for confirmation, while negative ratios below 0.1 were deemed appropriate for exclusion [12, 13]. Fagan plots were created for assumed pre-test probabilities of 25%, 50%, and 75% according to the bivariate Reitsma model, as described by Zwinderman et al. [14]. All analyses were conducted using R (version 4.3.2, R Foundation for Statistical Computing, Vienna, Austria), with “mada,” “meta” [15], “Metafor” [16], and “dmetatools” [17] packages.

## Results

### Screening and selection of articles

**Fig. 1:**
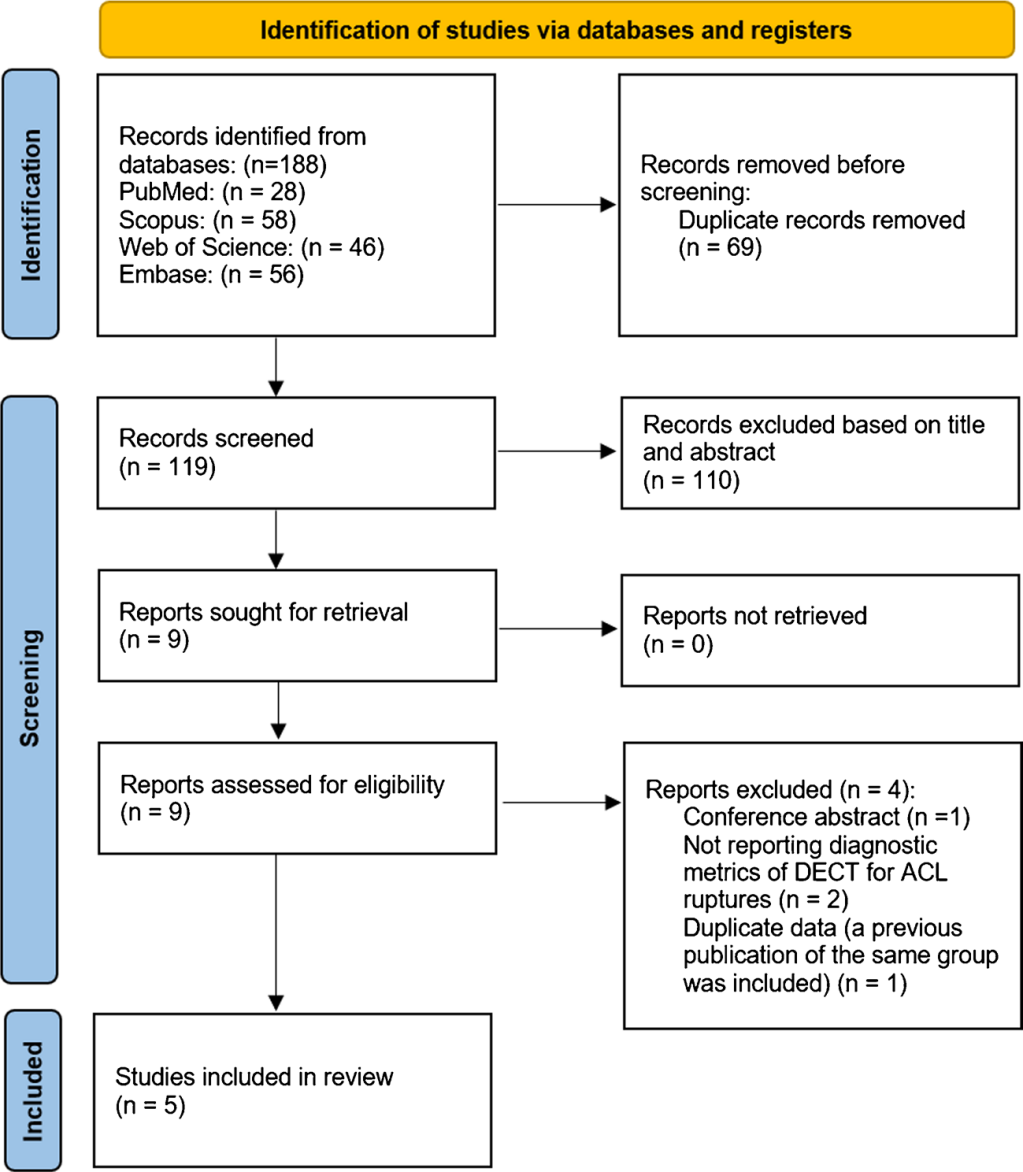
PRISMA flow diagram showing the review process. ACL anterior cruciate ligament, DECT dual-energy CT scan, PRISMA Preferred Reporting Items for Systematic Reviews and Meta-Analyses

We performed a systematic search of the literature following a predefined strategy, which initially yielded 188 articles. After eliminating duplicates, 119 papers were screened based on their titles and abstracts. At this stage, 110 articles were excluded. The full texts of the remaining nine papers were then reviewed in detail, resulting in the exclusion of 3 additional articles. A further study was removed [18] due to the overlapping sample with another study from the same group [19]. Ultimately, five articles met the inclusion criteria and were included in the study. The screening process and eligibility criteria adhered to PRISMA guidelines, as illustrated in Fig. 1.

### Study and patient characteristics

The meta-analysis included five studies assessing the performance of DECT in diagnosing ACL ruptures, involving a total of 191 patients with ACL injuries. Three studies focused exclusively on complete ACL ruptures [1, 2, 6], while the remaining two examined a combination of complete and partial ruptures [19, 20], with one study reporting DECT’s diagnostic performance for complete ruptures separately [19]. Four studies evaluated patients with acute/subacute injuries [2, 6, 19, 20], while one study included patients in the subacute/chronic phase [1]. Detailed patient characteristics are presented in Table 1. Image interpretation was primarily conducted by musculoskeletal radiologists, though in one of the included studies, an orthopedic surgeon also served as an interpreter [6]. MRI and/or arthroscopic evaluation were used as the reference tests in all studies. Further details on DECT examinations and their diagnostic performance are provided in Table 2.

**Table 1:** Demographics and characteristics of the included studies

First author, year	Country	Design	Sample size	Age	Female (%)	Type of injury	Staging of injury	Inclusion criteria
Bjorkman, 2022	Sweden	Prospective (cohort)	21 patients	Mean (range): 22 (15–37)	38.1	Partial or complete ACL rupture	Acute or subacute (within less than 6 weeks)	Patients suspected of ACL injury based on clinical/MRI findings within the past 6 weeks, without other significant knee injuries
Glazebrook, 2014	USA	Retrospective (case–control)	16 patients 11 controls	Patients Median (range): 24 (19–51) Controls Median (range): 36 (27–58)	Patients: 18.75 Controls: 36.36	Complete ACL rupture	Subacute or chronic	Patients with subacute or chronic unilateral traumatic complete ACL tears, confirmed by clinical evaluation and MRI ± arthroscopy Controls with no trauma history and clinically intact ACLs
Gruenewald, 2023	Germany	Retrospective (cohort)	85 patients	Mean ± SD (range): 44 ± 16 (14–89)	41.18	Complete ACL rupture (*n* = 11), partial ACL rupture (*n* = 4), avulsion fracture (*n* = 6)	Acute	Patients with ACL injury within less than 3 days, confirmed by MRI and/or arthroscopy within 2 weeks
Liu, 2023	China	Prospective (cohort)	51 patients	Mean ± SD (range): 27 ± 8.7 (15–47)	39.2	Complete ACL rupture	Acute or subacute	Patients with acute or subacute unilateral traumatic complete ACL tears, confirmed by clinical evaluation and MRI ± arthroscopy (Patients with concomitant meniscal or chondral lesions were also included)
Peltola, 2015	Finland	Retrospective (cohort)	18 patients	Mean (range): 36 (18–63)	55.6	Complete ACL rupture	Acute or subacute	Patients with ACL injury within less than 10 days, confirmed by MRI

*Abbreviations*: *ACL* anterior cruciate ligament, *MRI* magnetic resonance imaging, *SD* standard deviation

**Table 2:** Technical details and results of the included studies

First author, year	References test	Timing intervals	Radiation	Device model	Imaging technique	Slice thickness	Interpreter (experience)	Diagnostic performance
Bjorkman, 2022	Arthroscopy	Mean (range) Injury to DECT: 30 (7–55) d Injury to arthroscopy: 195 (15–55) d Injury to MRI: 31 d (47–349) d	CTDIvol: 4.0 mGy DLP: 87.8 mGycm	Dual-source CT scanner (Somatom Definition Force, Siemens Healthcare)	DECT with 80 kVp and 150 kVp tubes	N/A	A radiology resident (3 years) and an experienced MSK radiologist (more than 7 years)	Sen: 73.7%, Spe: 50%
Glazebrook, 2014	MRI + arthroscopy (*n* = 14) for patients with ACL tears	Median (range): Injury to MRI: 20 (1–90) d Injury to DECT: 59 (11–246) d MRI to DECT: 27 (7–242) d	CTDIvol: 17.2 mGy	Dual-source CT scanner in dual-energy mode (Somatom Definition Flash, Siemens Healthcare)	DECT with 80 kVp and 140 kVp tubes, DECT-based reconstruction with bone removal	0.75 mm	Four MSK radiologists (18, 7, 4, 3, and 2 years)	Sen: 81.3%, Spe: 90.9%
Gruenewald, 2023	MRI (*n* = 78) or arthroscopy (*n* = 34)	DECT to MRI/arthroscopy: 6 (0–13) d	N/A	Third-generation dual-source CT scanner in dual-energy mode (Somatom Force; Siemens Healthineers)	DECT with 90 kVp and 150 kVp tubes, DECT-based bone removal, and color-coded collagen reconstructions	1 mm	Five MSK radiologists (1 to 7 years)	Total Sen: 89.5%, Spe: 77.5% Complete ruptures Sen: 74.5%, Spe: 94.6%
Liu, 2023	MRI and arthroscopy (for the injured side)	Median (range): Injury to DECT: 2 (1–8.6) w MRI to DECT < 1 d	CTDIvol: 6.0 mGy DLP: 192 mGycm	Dual-source CT scanner in dual-energy mode (Somatom Drive; Siemens Healthcare)	DECT with 80 kVp and 140 kVp tubes, soft tissue reconstruction kernel, and bone marrow–specific highlighting kernel to highlight ACL	0.75 mm	An orthopedic surgeon (14 years) and an MSK radiologist (11 years)	Sen: 98%, Spe: 98%
Peltola, 2015	MRI	Mean (Range): Injury to DECT: 1.6 (1–10) d Injury to MRI: 4.6 (0–25) d	DLP: 603 ± 83 mGycm	GE Discovery- CT750 HD	DECT with 80 kVp and 140 kVp tubes, automatic bone removal, and collagen mapping	0.33 mm	Two MSK radiologists (2 and over 10 years)	Sen: 83.3%, Spe: 100%

*Abbreviations*: *ACL* anterior cruciate ligament, *CT* computed tomography, *CTDIvol* CT dose index volume, *d* days, *DECT* dual-energy CT scan, *DLP* dose length product, *kVp* Kilovoltage peak, *MRI* magnetic resonance imaging, *MSK* musculoskeletal, *N/A* no answer, *SD* standard deviation, *Sen* sensitivity, *Spe* specificity, *w* weeks

### Diagnostic findings of DECT

**Fig. 2:**
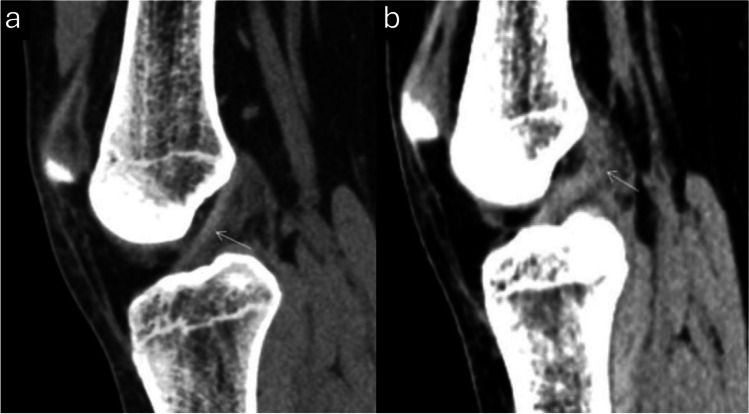
Oblique sagittal mixed kV DECT image showing an intact anterior cruciate ligament (ACL) (arrow) in a single slice through the intercondylar notch (**a**). Oblique sagittal mixed kV DECT image of a 33-year-old woman with a complete ACL tear, scanned 37 days post-injury, which shows the torn ACL (arrow) in the intercondylar notch (**b**). Adapted from the cited study with reproduction permission [1]

**Fig. 3:**
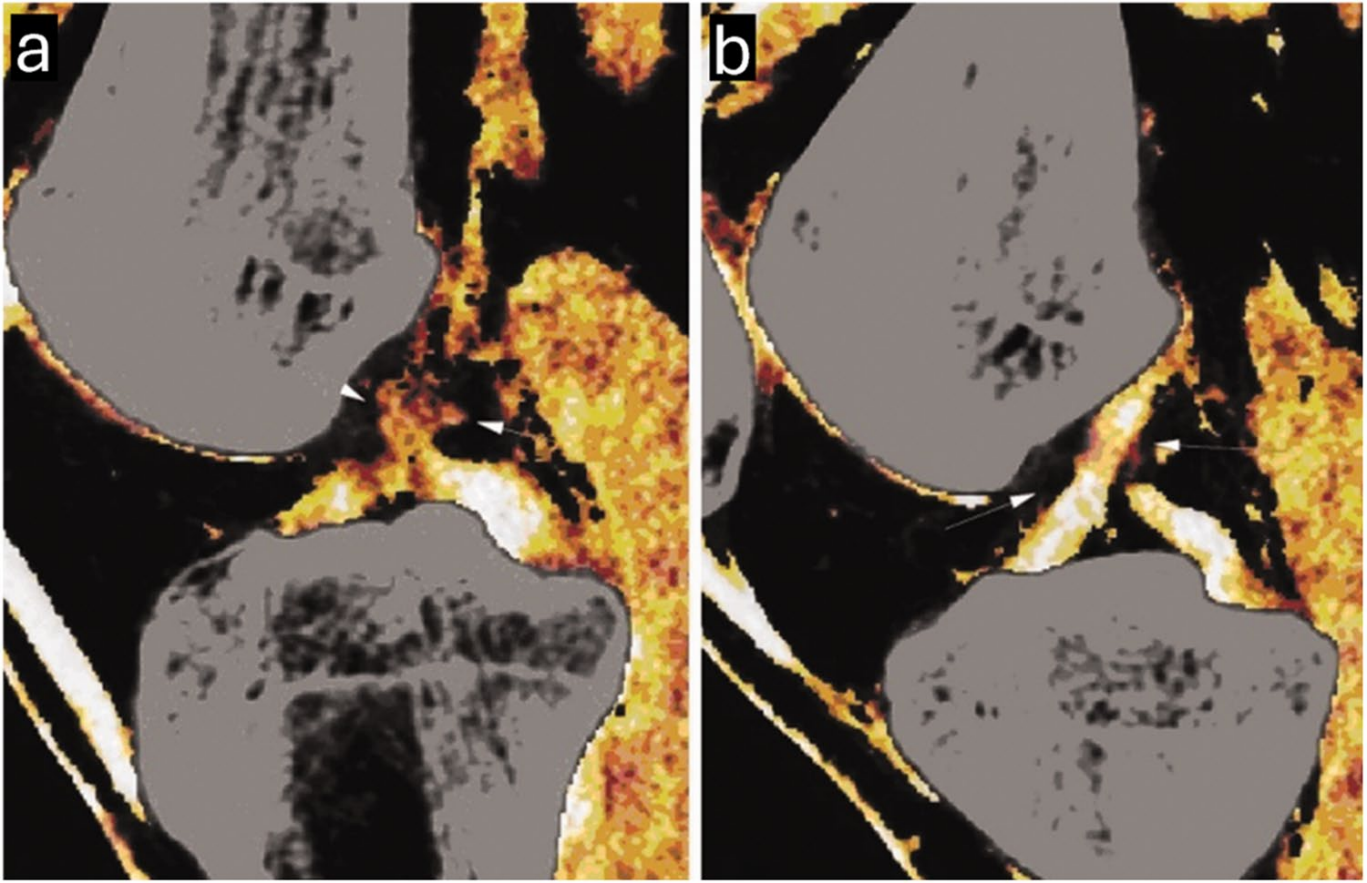
Color mapping of the left anterior cruciate ligament (ACL) tear in a 24-year-old man, confirmed by arthroscopy. **a** Torn left ACL. **b** Intact right ACL. Adapted from the cited study, published under a Creative Commons License [21]

**Fig. 4:**
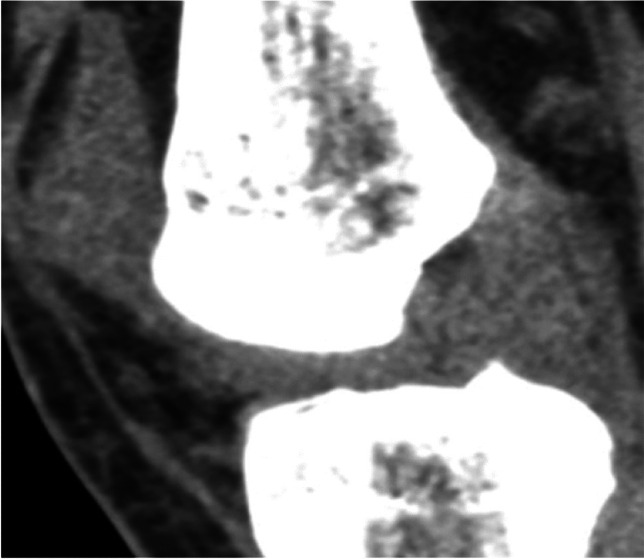
False-positive DECT finding in a 27-year-old man, where DECT suggested an ACL tear, but arthroscopy confirmed it was intact. Adapted from the cited study, published under a Creative Commons License [20]

The included studies anatomically evaluated the ACL and considered an intact ACL with continuous fibers as normal, whereas an ACL with discontinuous, separated, or totally avulsed fibers was considered abnormal [1, 2, 6, 19, 20]. Figure 2 illustrates examples of both an intact and a torn ACL on mixed kV grayscale DECT images [1]. Additionally, an abnormal orientation of the ACL (less steep than the roof of the intercondylar notch, also known as Blumensaat’s line) was regarded as an indication of a complete rupture [18]. The presence of joint effusion was also considered a marker of damage to the knee’s internal structures [18]. In addition, Gruenewald et al. used an experimental algorithm for color-coded collagen reconstruction and found that it increased the diagnostic accuracy for evaluating cruciate ligament integrity in patients with acute trauma when compared to traditional grayscale CT imaging [19]. Figure 3 represents an example of color-coded collagen mapping of the ACL [21]. Moreover, Fig. 4 presents a case of a false-positive DECT finding, highlighting potential limitations in DECT specificity when compared to arthroscopy [20].

### Quality assessment

The methodological quality of the incorporated studies is detailed in Table 3. Among the five reviewed studies, significant risk of bias (ROB) concerns or unclarities were noted in 4 studies, especially regarding selection bias concerns and patient flow considerations.

**Table 3:** Methodological quality assessment of the included studies

Author	Q1	Q2	Q3	Q4	Q5	Q6	Q7
Bjorkman et al., 2022	Low	Low	Low	Low	Low	Low	Unclear
Glazebrook et al. 2014	Unclear	Low	Low	Low	Low	Low	Unclear
Gruenewald et al., 2023	Unclear	Low	Low	Low	Low	Low	Unclear
Liu et al., 2024	Low	Low	Low	Low	Low	Low	Low
Peltola et al., 2015	High	Unclear	Low	Low	Low	Low	High

*Q* question

Q1: Could the selection of patients have introduced bias?

Q2: Are there concerns that the included patients do not match the review question?

Q3: Could the conduct or interpretation of the index test have introduced bias?

Q4: Are there concerns that the index test, its conduct, or its interpretation differ from the review question?

Q5: Could the reference standard, its conduct, or its interpretation have introduced bias?

Q6: Are there concerns that the target condition as defined by the reference standard does not match the review question?

Q7: Could the patient flow have introduced bias?

The methodological quality of the included studies is summarized in Table 3. Notably, 4 out of the five studies reviewed exhibited significant concerns or uncertainties related to the risk of bias [1, 2, 19, 20], particularly in areas such as selection bias and patient flow.

### Overall meta-analysis assessing the performance of DECT in detecting ACL ruptures

**Fig. 5:**
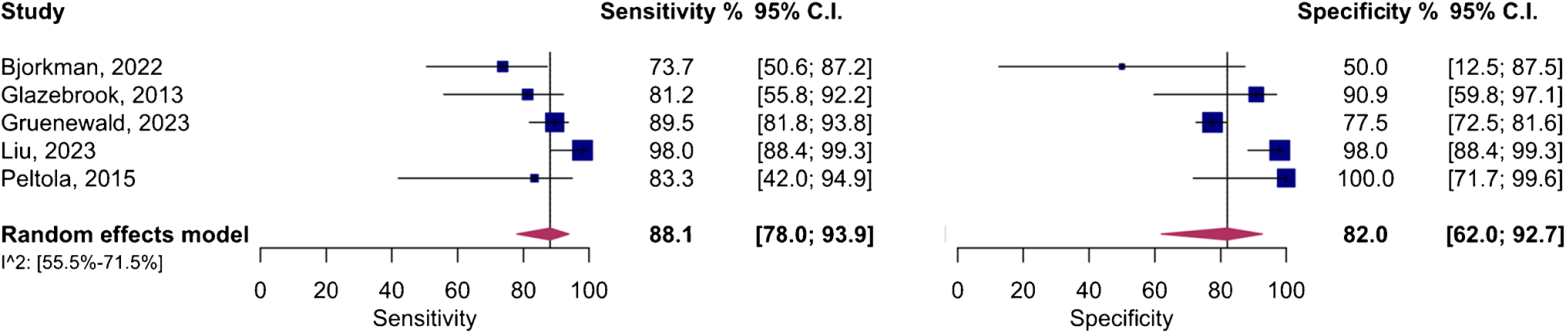
Forest plot and summary statistics of the diagnostic test accuracy (DTA) meta-analysis incorporating all included studies. CI confidence interval

**Fig. 6:**
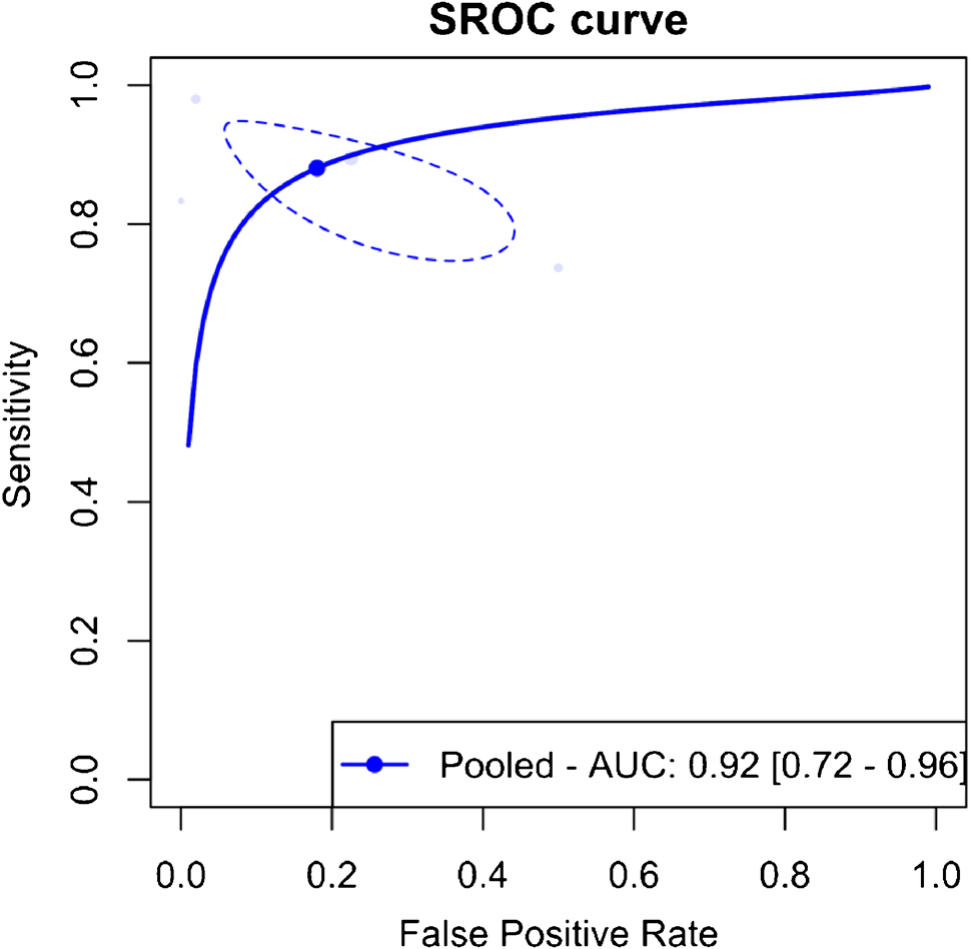
Summary receiver operating characteristic (SROC) curve for the diagnostic test accuracy (DTA) meta-analysis encompassing all included studies. AUC area under the curve, SROC summary receiver operating characteristic

**Fig. 7:**
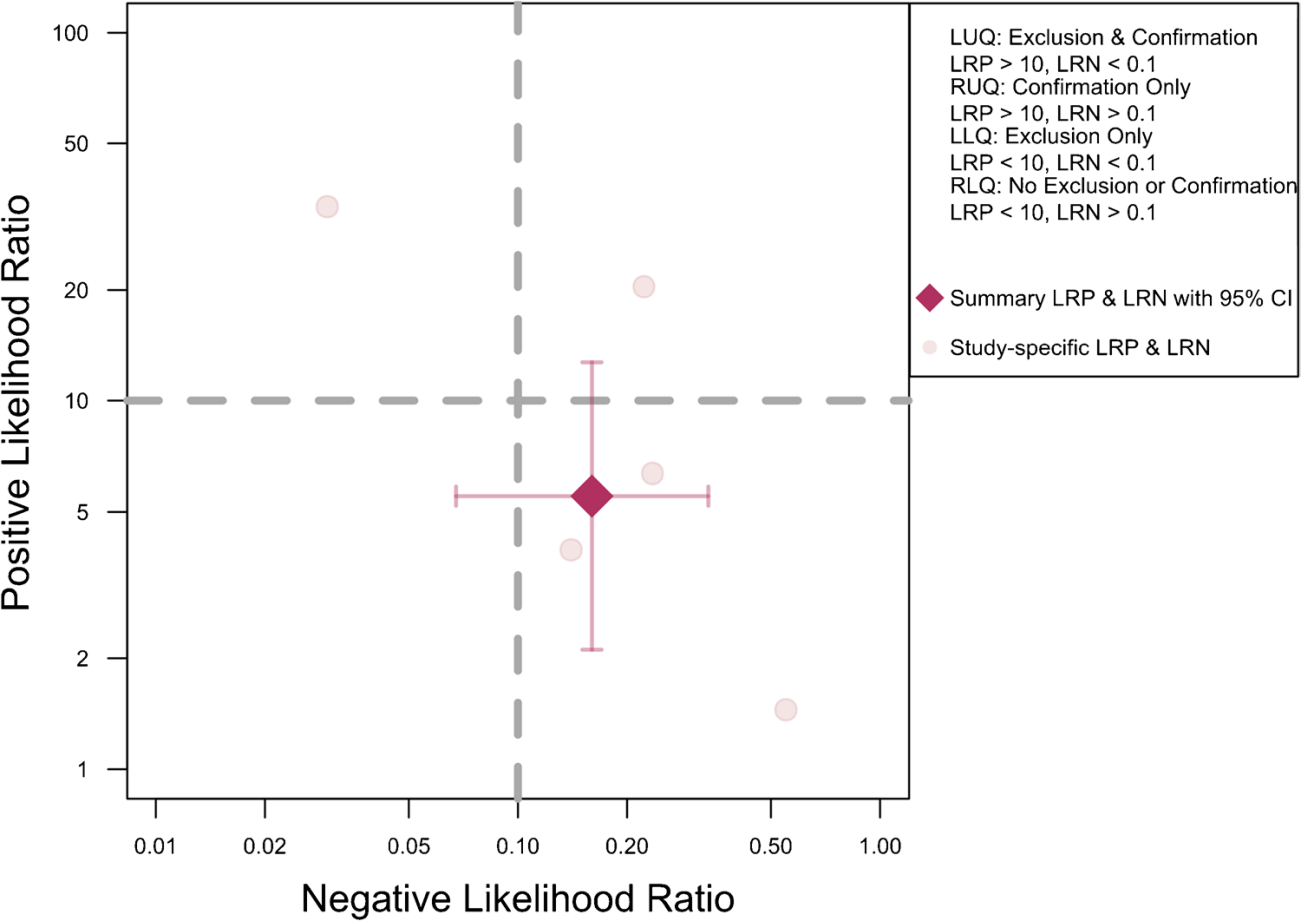
Likelihood ratio scattergram of included studies indicating that the performance is suboptimal for both exclusion and confirmation purposes. LLQ left lower quadrant, LRN likelihood ratio, negative, LRP likelihood ratio, positive, LUQ left upper quadrant, RLQ right lower quadrant, RUQ right upper quadrant

**Fig. 8:**
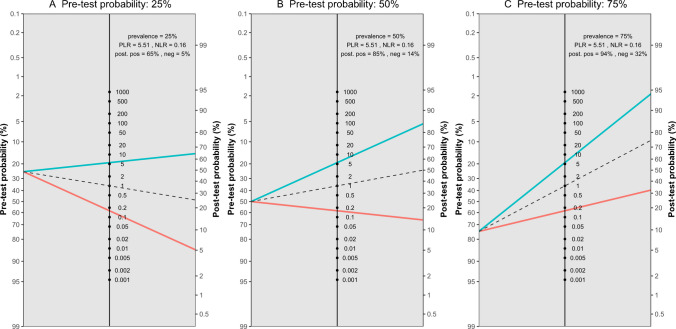
Fagan plot analysis utilizing summary positive and negative likelihood ratio results from the meta-analysis of all included studies, considering hypothetical pre-test probabilities of 25%, 50%, and 75%. PLR positive likelihood ratio, NLR negative likelihood ratio, Neg negative, Pos positive

The meta-analysis of five studies evaluating the accuracy of DECT in diagnosing ACL ruptures showed pooled sensitivity and specificity of 88.1% (95% CI, 78.0–93.9%) and 82.0% (95% CI, 62.0–92.7%), respectively, as depicted in Fig. 5. The AUC for the SROC curve was 0.92 (95% CI, 0.72–0.96), illustrated in Fig. 6. Figure 7 displays a scattergram of positive and negative likelihood ratios, suggesting suboptimal performance for both exclusion and confirmation purposes. In the Fagan plot study, with pre-test probabilities of 25%, 50%, and 75% for ACL ruptures, the positive post-test probabilities are 65%, 85%, and 94%, while the negative post-test probabilities are 5%, 14%, and 32%, respectively (Fig. 8).

The meta-analysis also revealed significant heterogeneity, as shown in Fig. 5. An influential analysis was conducted to identify potential outliers, revealing the study by Liu et al. [6] as a significant outlier. After excluding this outlier, the meta-analysis of the remaining four studies showed pooled sensitivity and specificity of 83.2% (95% CI, 73.7–89.7%) and 77.9% (95% CI, 73.2–82.0%), with low heterogeneity, as shown in Supplementary Fig. 1. The AUC for the SROC curve was 0.79 (95% CI, 0.75–0.94), illustrated in Supplementary Fig. 2. Supplementary Fig. 3 displays a scattergram of positive and negative likelihood ratios, and Supplementary Fig. 4 represents the Fagan plot study after exclusion on the outlier study.

### Performance of DECT in detecting complete ruptures of ACL

**Fig. 9:**
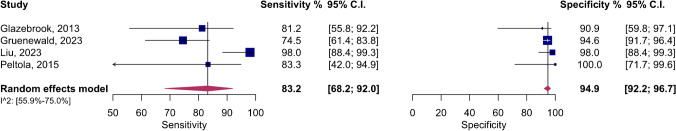
Forest plot and summary statistics of the diagnostic test accuracy (DTA) meta-analysis of studies including cases with complete rupture of anterior cruciate ligament. CI confidence interval

**Fig. 10:**
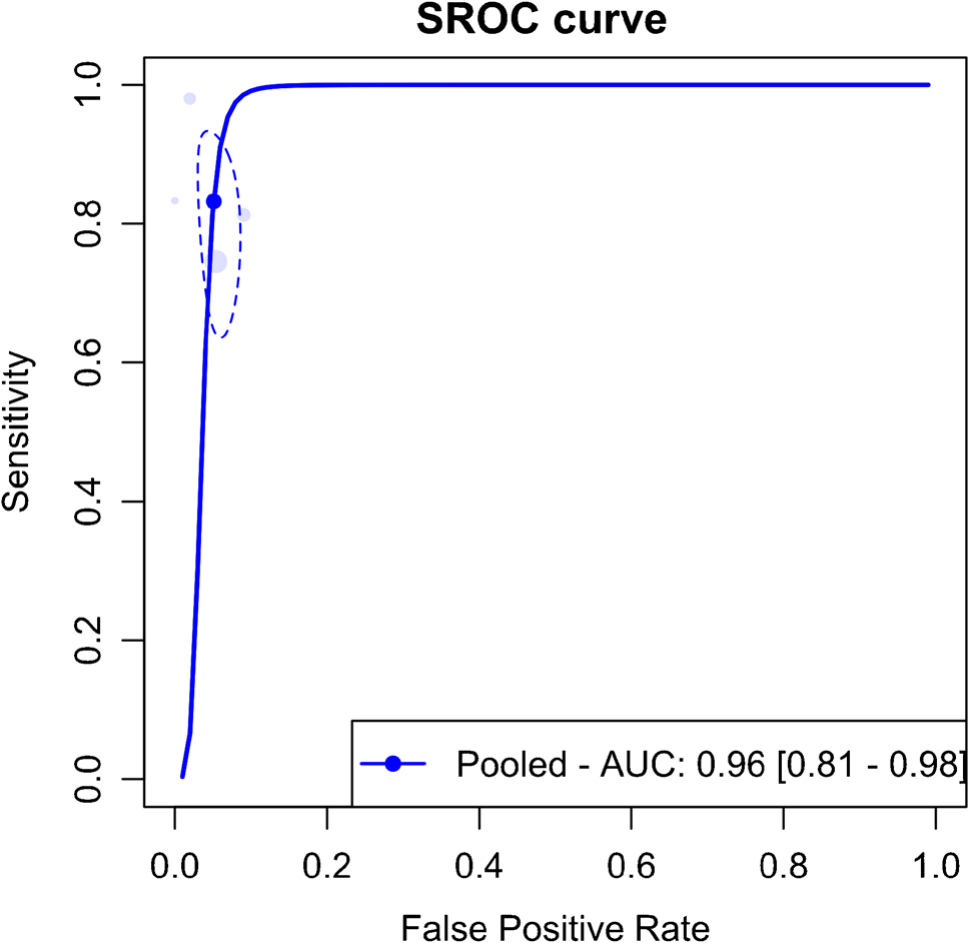
Summary receiver operating characteristic curve (SROC) for the diagnostic test accuracy (DTA) meta-analysis of studies, including cases with complete rupture of the anterior cruciate ligament. AUC area under the curve; SROC summary receiver operating characteristic

Four of the included studies provided performance metrics for DECT in identifying complete ACL ruptures. The meta-analysis of these studies showed pooled sensitivity and specificity of 83.2% (95% CI, 68.2–92.0%) and 94.9% (95% CI, 92.2–96.7%), respectively, as shown in Fig. 9. The AUC for the SROC curve was 0.96 (95% CI, 0.81–0.98), illustrated in Fig. 10.

**Fig. 11:**
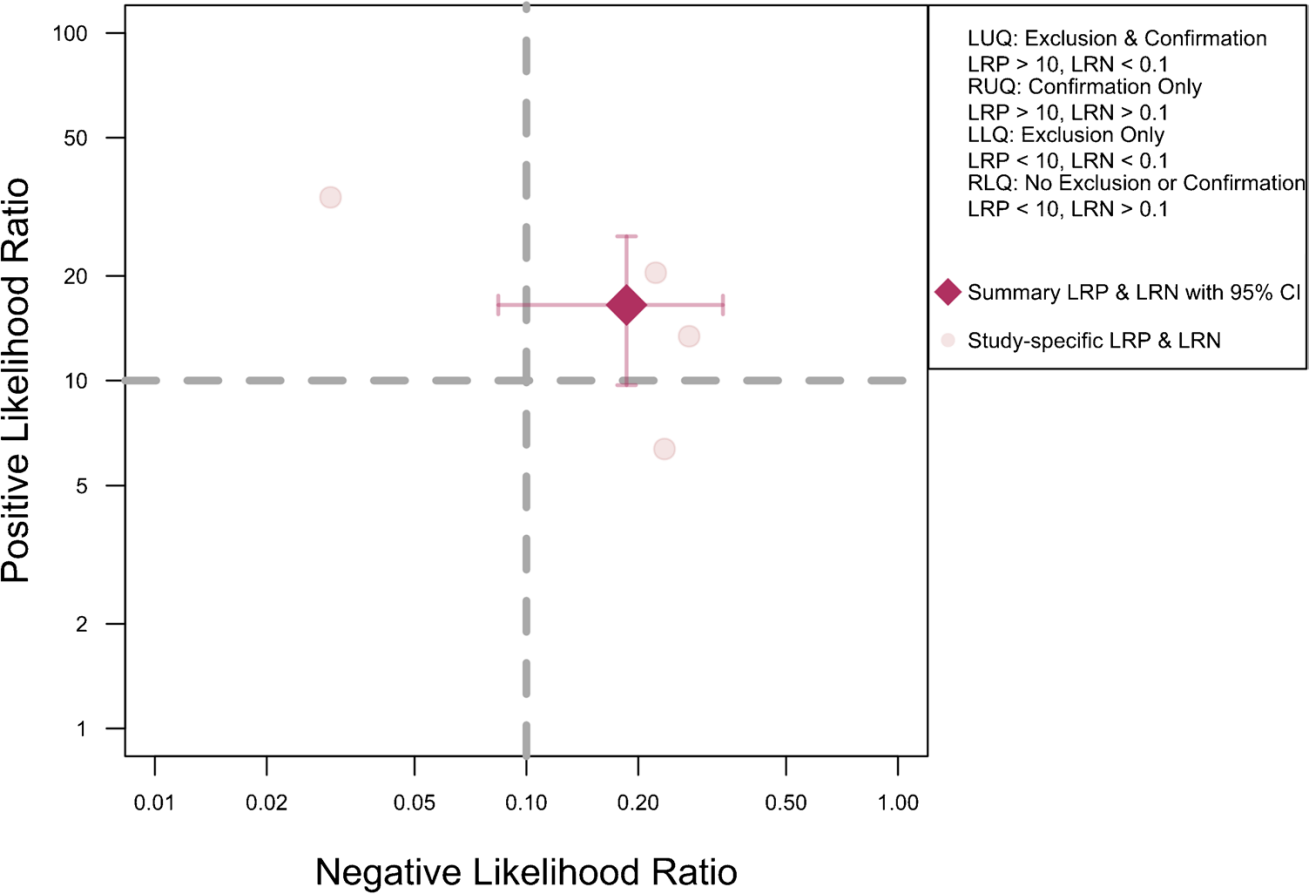
Likelihood ratio scattergram of studies including cases with complete rupture of anterior cruciate ligament, indicating that the performance is optimal for only confirmation purposes. LLQ left lower quadrant, LRN likelihood ratio, negative, LRP likelihood ratio, positive, LUQ left upper quadrant, RLQ right lower quadrant, RUQ right upper quadrant

**Fig. 12:**
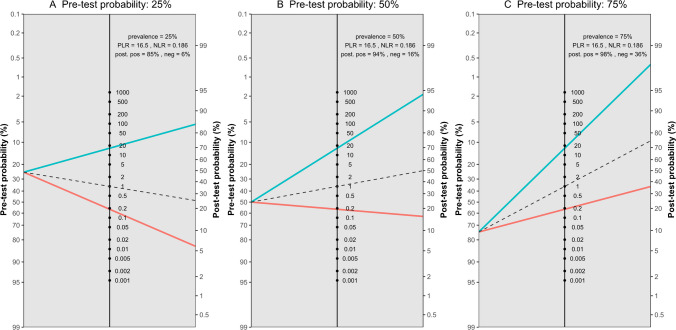
Fagan plot analysis utilizing summary positive and negative likelihood ratio results from the meta-analysis of studies including cases with complete rupture of anterior cruciate ligament, considering hypothetical pre-test probabilities of 25%, 50%, and 75%. PLR positive likelihood ratio, NLR negative likelihood ratio, Neg negative, Pos positive

Figure 11 presents a scattergram of positive and negative likelihood ratios, indicating that DECT is optimal for confirmation but not suitable for exclusion. In the Fagan plot study, considering pre-test probabilities of 25%, 50%, and 75% for ACL ruptures, the positive post-test probabilities are 85%, 94%, and 98%, while the negative post-test probabilities are 6%, 16%, and 36%, respectively (Fig. 12).

The meta-analysis showed significant heterogeneity, as depicted in Fig. 9. To identify potential outliers, an influential analysis was performed, which pinpointed the study by Liu et al. [6] as a significant outlier. Excluding this outlier, the meta-analysis of the remaining three studies revealed pooled sensitivity and specificity of 75.5% (95% CI, 64.9–83.7%) and 94.2% (95% CI, 91.4–96.2%), with minimal heterogeneity, as shown in Supplementary Fig. 5. The AUC for the SROC curve after excluding the outlier was 0.79 (95% CI, 0.75–0.94), depicted in Supplementary Fig. 6. Supplementary Fig. 7 displays a scattergram of positive and negative likelihood ratios, and Supplementary Fig. 8 represents the Fagan plot study after removing the outlier.

### Performance of DECT in detecting ruptures of ACL in acute/subacute settings

**Fig. 13:**
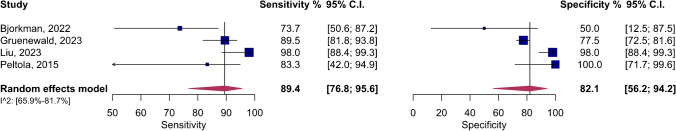
Forest plot and summary statistics of the diagnostic test accuracy (DTA) meta-analysis of studies on anterior cruciate ligament rupture in acute/subacute setting. CI confidence interval

**Fig. 14:**
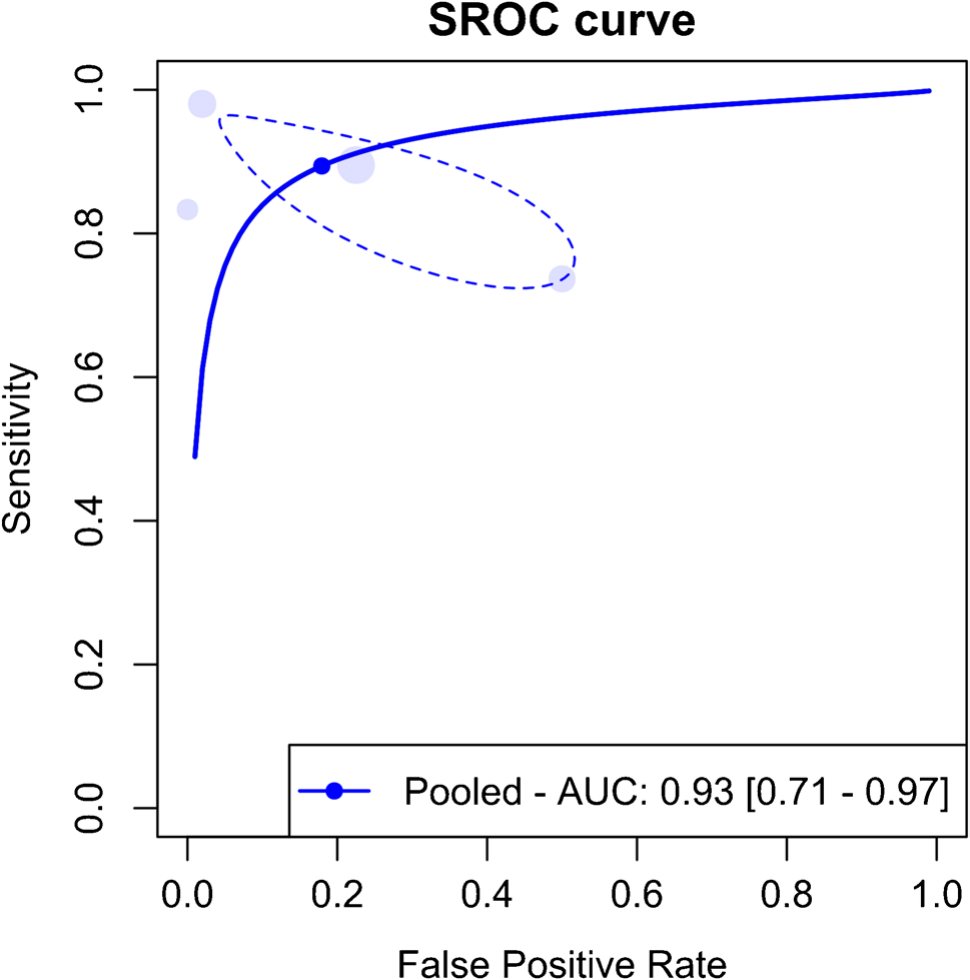
Summary receiver operating characteristic curve (SROC) for the diagnostic test accuracy (DTA) meta-analysis of studies on anterior cruciate ligament rupture in acute/subacute setting. AUC area under the curve, SROC summary receiver operating characteristic

Four of the included studies assessed DECT’s performance in detecting ACL ruptures in the acute/subacute stages. The meta-analysis results indicated pooled sensitivity and specificity of 89.4% (95% CI, 76.8–95.6%) and 82.1% (95% CI, 56.2–94.2%), respectively, as shown in Fig. 13. The AUC for the SROC curve was 0.93 (95% CI, 0.71–0.97), depicted in Fig. 14.

**Fig. 15:**
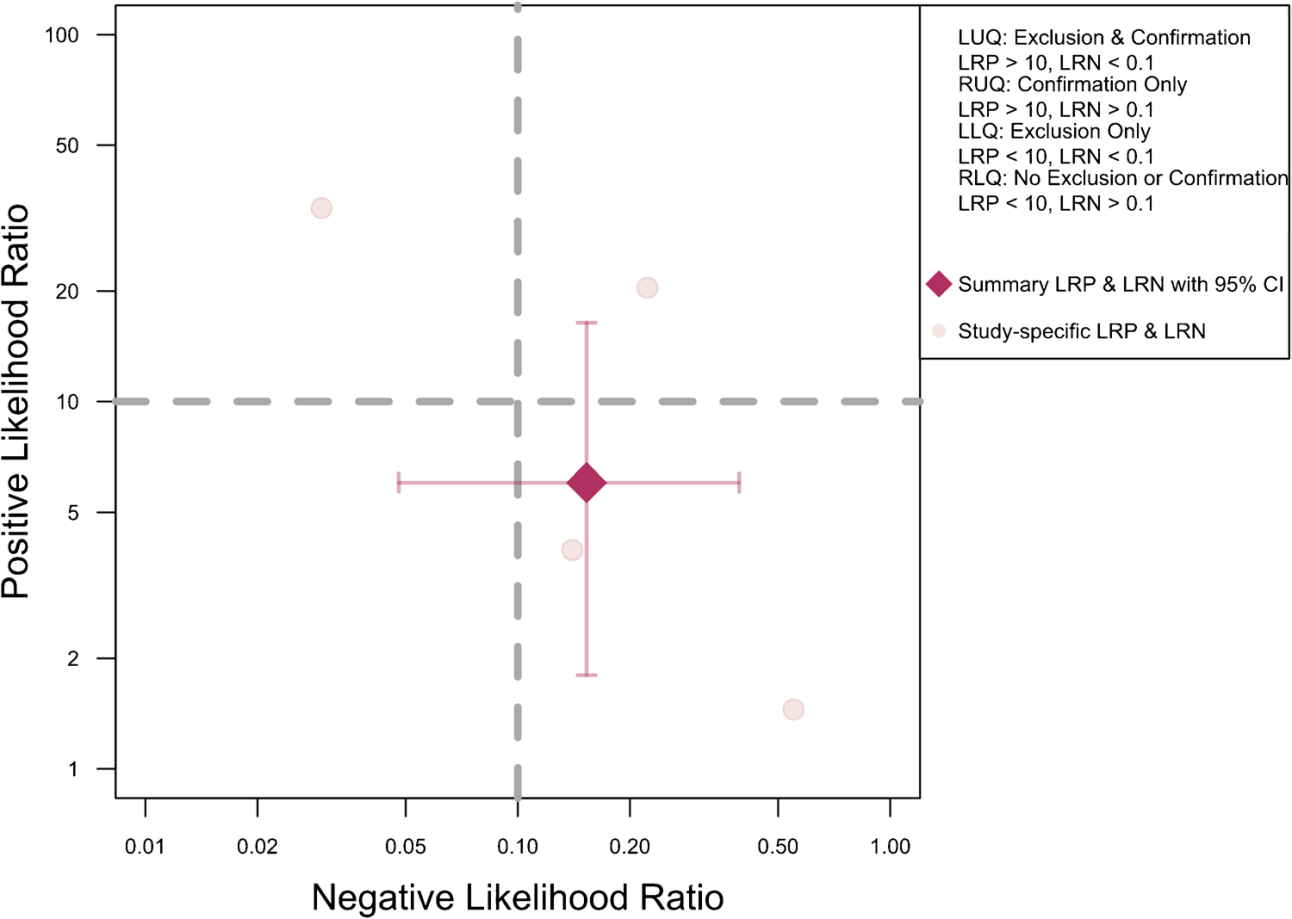
Likelihood ratio scattergram of studies on anterior cruciate ligament rupture in acute/subacute setting, indicating that the performance is suboptimal for both exclusion and confirmation purposes. LLQ left lower quadrant, LRN likelihood ratio, negative, LRP likelihood ratio, positive, LUQ left upper quadrant, RLQ right lower quadrant, RUQ right upper quadrant

**Fig. 16:**
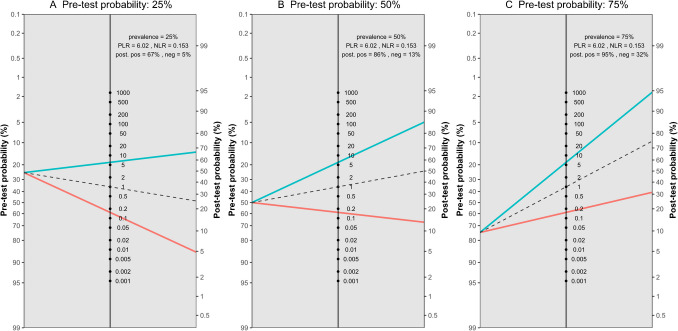
Fagan plot analysis utilizing summary positive and negative likelihood ratio results from the meta-analysis of studies on anterior cruciate ligament rupture in acute/subacute setting, considering hypothetical pre-test probabilities of 25%, 50%, and 75% for ACL rupture. PLR positive likelihood ratio, NLR negative likelihood ratio, Neg negative, Pos positive

Figure 15 displays a scattergram of positive and negative likelihood ratios, indicating suboptimal performance for both exclusion and confirmation. In the Fagan plot study, with pre-test probabilities of 25%, 50%, and 75% for ACL ruptures, the positive post-test probabilities were 65%, 85%, and 94%, and the negative post-test probabilities were 5%, 14%, and 32%, respectively (Fig. 16).

The meta-analysis identified significant heterogeneity, as shown in Fig. 13. An influential analysis identified Liu et al. [6] as a significant outlier. After excluding this outlier, the meta-analysis of the remaining three studies showed pooled sensitivity and specificity of 83.8% (95% CI, 72.7–91.0%) and 77.2% (95% CI, 72.3–81.5%), with some heterogeneity depicted in Supplementary Fig. 9. The AUC for the SROC curve was 0.83 (95% CI, 0.68–0.92), shown in Supplementary Fig. 10. Supplementary Fig. 11 shows a scattergram of positive and negative likelihood ratios, and Supplementary Fig. 12 presents the Fagan plot study after excluding the outlier.

## Discussion

Our meta-analysis included five studies to investigate the diagnostic accuracy of DECT in diagnosing complete or partial ACL rupture [1, 2, 6, 19, 20]. The meta-analysis for ACL rupture diagnosis revealed pooled sensitivity, specificity, and AUC of 88.1% (95% CI, 78.0–93.9%), 82% (95% CI, 62.0–92.7%), and 0.92 (95% CI, 0.72–0.96), respectively. Our meta-analysis showed significant heterogeneity between included studies; this can be caused by methodological variations, such as the staging of injury by time and severity and differences in inclusion and exclusion criteria. The results indicate that DECT has the potential to be seen as a valuable diagnostic tool for ACL injuries.

Various subgroup analyses also demonstrated that DECT possesses a high diagnostic accuracy for ACL injuries, with consistent findings supporting its usefulness as an effective tool for evaluating knee joint injuries. Our study revealed the potential of DECT in diagnosing complete ACL tears, with a pooled sensitivity and specificity of 83.2% (95% CI, 68.2–92.0%) and 94.9% (95% CI, 92.2–96.7%), respectively. Also, the meta-analysis on the utility of DECT in acute/subacute ACL injury settings yielded pooled sensitivity and specificity of 89.4% (95% CI, 76.8–95.6%) and 82.1% (95% CI, 56.2–94.2%), respectively. These results highlight that DECT shows high sensitivity and specificity, which allows the clinician to diagnose ACL ruptures precisely and rule out negative cases. High specificity, especially in the early stages of assessment, can be beneficial since it helps the clinician avoid unnecessary diagnostic tests and treatment procedures, reducing the associated costs and risks.

MRI is the most commonly used noninvasive diagnostic modality for diagnosing ACL injuries. Compared to arthroscopy, which is known as the best diagnostic test for ACL injuries, MRI has a sensitivity of 86–95% and specificity of 50–100% [22–26]. Other imaging modalities, such as DECT, have been evaluated in comparison with MRI. DECT has enhanced musculoskeletal imaging, providing superior precision compared to single-energy CT [27, 28]. Beyond traditional monoenergetic attenuation–based CT imaging, advanced techniques like photon-counting CT and spectral CT are now employed for material decomposition by leveraging the distinct behavior of materials at different energy levels using specialized post-processing algorithms for enhanced diagnostic accuracy [29, 30]. As a subtype of spectral acquisitions, DECT typically uses low (80–90 kV) and high (140–150 kV) energy spectra to differentiate tissues based on their varying attenuation coefficients in these energy levels. This differentiation relies on the photoelectric effect, influenced by X-ray energy and tissue atomic number, and the Compton effect, related to tissue electronic density [27, 31].

In ligament imaging, DECT’s ability to distinguish collagenous structures from surrounding tissues is particularly advantageous. Collagen, the primary component of ligaments, has unique dual-energy index values due to its densely packed structure and specific side chains, such as hydroxylysine and hydroxyproline [32]. These indices are calculated based on the differences in X-ray absorption between the low- and high-energy spectra, reflecting the tissue’s atomic composition and density. In DECT imaging, these indices are used in a three-material decomposition algorithm that separates collagen from fat and soft tissue. The two energy datasets are processed in a multimodality workstation, where collagen can also be color-coded (e.g., orange or yellow) for clearer visualization [28, 32, 33]. By using these indices, DECT provides an enhanced evaluation of ligamentous injuries.

Various DECT acquisition methods, such as dual-source and fast kilovolt switching, provide high spatial resolution, even down to 0.5–1 mm, surpassing MRI’s typical 2–3 mm resolution [34–37]. This improved resolution is especially beneficial for assessing large ligaments like ACL. Several post-processing techniques for DECT may further improve its diagnostic accuracy. These include material decomposition, tendon-specific color mapping, collagen mapping, and bone removal (virtual non-calcium or VNCa, which allows for bone marrow edema/contusion detection), all of which leverage the differences in attenuation gradients of various elements and their variation between the two energy levels used in DECT [38, 39]. Gruenewald et al. explored an experimental post-processing technique involving a color-coded collagen reconstruction algorithm. They observed that incorporating this method into conventional grayscale CT enhanced diagnostic confidence [19]. Still, the effectiveness of these post-processing methods in enhancing DECT’s diagnostic accuracy for ACL injuries remains debated, partly due to variations in timing and study populations. Importantly, while tendon-specific mapping for evaluating tendon integrity was the area of focus in the ACL-specific studies included in this review, VNC techniques are also recognized for their ability to suggest ACL injuries by providing diagnostic clues about the location of bone injury and the mechanism of injury, as indicated by patterns of bone contusion [40, 41].

Tissue properties change at different stages of injury, meaning that each method may be more suitable for specific stages of the disease process. In acute settings, for instance, severe edema in the damaged ACL may impair diagnostic accuracy, highlighting the need to optimize DECT parameters for better visualization of complex tissue characteristics in acute knee injuries [20, 42]. Although bone edema poses diagnostic challenges, DECT indices can also be used to detect bone marrow edema in the origin and insertion of ligaments, as well as contusion patterns, which can serve as a diagnostic clue in cases where direct visualization of ligament disruption is not possible. In addition to detecting bone marrow edema, DECT VNC material decomposition identifies specific bone contusion patterns, such as coup-countercoup kissing contusions, which are indicative of hyperextension and pivot shift mechanisms, both of which are suggestive of ACL injury [40, 43, 44]. This capability adds an additional layer to DECT’s utility in diagnosing ligamentous injuries, especially when primary signs are not easily identified [27, 28, 45].

Bjorkman et al. evaluated the performance of DECT in patients with suspected ACL injury and reported that, compared to MRI, the diagnostic accuracy of DECT in detecting bone marrow lesions in subacute knee injury was moderate [41]. Furthermore, de Bakker et al. studied edema-like marrow signal intensity associated with acute knee injury and found out that, compared to MRI, DECT obtained less sensitive results. However, they suggested that the pattern of edema-like marrow signal intensity can be used as an indicator of ligamentous injury and highlight the need for further follow-up imaging [45]. In another study, Koskinen et al. revealed that, compared to MRI, DECT underestimates the bone marrow edema volume in the tibia but not in the femur [46].

Consequently, DECT is gaining popularity, especially in the field of musculoskeletal imaging; many studies have recently explored the potential of DECT in diagnosing muscle and ligament injury [47]. Compared to MRI, this method has better availability, faster acquisition, and is more cost-effective. It is also less prone to motion and metal-related artifacts compared to MRI [48, 49]. DECT can also be of more use in trauma management settings, as various post-processing methods for ACL differentiation are available for DECT and can act as a substitute imaging method in situations when MRI is contraindicated or unavailable [50]. By accelerating diagnosis and treatment in patients with a high clinical suspicion of ACL injury, patients can experience reduced immobilization time and have better treatment outcomes.

The findings of the present review should be interpreted considering certain limitations. A notable limitation of the present review is that many of the included studies used MRI as the reference standard for diagnosing ACL injuries. MRI is not an ideal gold standard in this context and has a significantly lower diagnostic performance compared to arthroscopy [51]. Future studies should focus on comparing DECT directly with arthroscopy, which remains the definitive diagnostic method for ACL injuries [52], to more accurately determine DECT's diagnostic performance.

Another limitation is the scarcity of research in this field, which restricts the ability to draw definitive conclusions regarding the accuracy of DECT in diagnosing ACL injuries. Also, significant heterogeneity was present due to variations in study protocols, study populations, timing, and injury severity among the included patients. However, due to the limitations in the available literature, a comprehensive investigation into all potential sources of this variability was not feasible. Additional studies are necessary to validate these results. Furthermore, none of the included studies evaluated patients with prior ACL reconstruction. Therefore, further research is necessary to determine the utility of DECT in detecting reinjury in this specific population.

Our findings indicate that DECT can be a valuable diagnostic tool for ACL injuries, particularly as an adjunct or when conventional MRI is contraindicated or unavailable. Its use enables timely diagnosis and accurate localization of the injury, offering an effective alternative in appropriate clinical settings.

## Supplementary Information

Below is the link to the electronic supplementary material.

Supplementary file1 (DOCX 3418 KB)

## Data Availability

The datasets analyzed during the current study are available from the corresponding author upon reasonable request. Due to the nature of a meta-analysis, individual participant data was not collected directly, and all source data were extracted from previously published studies, which are cited within this manuscript.
