# Supplementary material for: Diagnostic performance of dual-energy computed tomography in detecting anterior cruciate ligament injuries: a systematic review and meta-analysis
Source: Skeletal Radiol. 2024 Nov 21;54(6):1247–62. doi: 10.1007/s00256-024-04833-x (PMC12000185; doi:10.1007/s00256-024-04833-x)
Supplement: Supplementary file 1 — Supplementary file1 (DOCX 3418 KB) [file 256_2024_4833_MOESM1_ESM.docx]

| **Supplementary table 1. Search strategy for four databases** | | |
| --- | --- | --- |
| **Database** | **Search strategy** | **Results** |
| PubMed | (((("dual energy CT") OR ("dual-energy CT") OR ("dual energy computed tomography") OR ("dual energy computed-tomography") OR ("dual-energy computed tomography") OR ((computed tomography, x ray[MeSH Terms]) AND (dual-energy)) OR ((Tomography, X-Ray, computed[MeSH Terms]) AND ("dual energy")) OR (DECT) OR (DE-CT)) OR ("collagen map*")))  AND  (("Posterior Cruciate Ligament") OR (PCL) OR ("Anterior Cruciate Ligament") OR ("ACL") OR ("Patellar Ligament") OR ("Medial Collateral Ligament") OR ("MCL") OR ("lateral Collateral Ligament") OR ("LCL") OR ("Knee injury") OR ("Knee trauma") OR ("knee ligament*") OR ("cruciate ligament") OR ("ligament rupture") OR ("Posterior Cruciate Ligament"[Mesh] OR "Anterior Cruciate Ligament"[Mesh] OR "Patellar Ligament"[Mesh] OR "Medial Collateral Ligament, Knee"[Mesh]) OR ("ligamentous knee") OR ("Cruciate Ligament*") OR ("ACL tear") OR ("ACL rupture")) | 28 |
| Scopus | (TITLE-ABS-KEY("dual energy CT") OR TITLE-ABS-KEY("dual-energy CT") OR TITLE-ABS-KEY("dual energy computed tomography") OR TITLE-ABS-KEY("dual energy computed tomography") OR TITLE-ABS-KEY("dual-energy computed tomography") OR TITLE-ABS-KEY(DECT) OR TITLE-ABS-KEY(DE-CT) OR TITLE-ABS-KEY("collagen map*")) AND (TITLE-ABS-KEY("Posterior Cruciate Ligament") OR TITLE-ABS-KEY(PCL) OR TITLE-ABS-KEY("Anterior Cruciate Ligament") OR TITLE-ABS-KEY(ACL) OR TITLE-ABS-KEY("Patellar Ligament") OR TITLE-ABS-KEY("Medial Collateral Ligament") OR TITLE-ABS-KEY(MCL) OR TITLE-ABS-KEY("lateral Collateral Ligament") OR TITLE-ABS-KEY(LCL) OR TITLE-ABS-KEY("Knee injury") OR TITLE-ABS-KEY("Knee trauma") OR TITLE-ABS-KEY("knee ligament*") OR TITLE-ABS-KEY("cruciate ligament") OR TITLE-ABS-KEY("ligament rupture") OR TITLE-ABS-KEY("ligamentous knee") OR TITLE-ABS-KEY("cruciate ligament*") OR TITLE-ABS-KEY("ACL tear") OR TITLE-ABS-KEY("ACL rupture")) | 58 |
| Web of sciences | (ALL=("dual energy CT") OR ALL=("dual-energy CT") OR ALL=("dual energy computed tomography") OR ALL=("dual energy computed tomography") OR ALL=("dual-energy computed tomography") OR ALL=(DECT) OR ALL=(DE-CT) OR ALL=("collagen map*")) AND (ALL=("Posterior Cruciate Ligament") OR ALL=(PCL) OR ALL=("Anterior Cruciate Ligament") OR ALL=(ACL) OR ALL=("Patellar Ligament") OR ALL=("Medial Collateral Ligament") OR ALL=(MCL) OR ALL=("lateral Collateral Ligament") OR ALL=(LCL) OR ALL=("Knee injury") OR ALL=("Knee trauma") OR ALL=("knee ligament*") OR ALL=("cruciate ligament") OR ALL=("ligament rupture") OR ALL=("ligamentous knee") OR ALL=("cruciate ligament*") OR ALL=("ACL tear") OR ALL=("ACL rupture")) | 46 |
| Embase | 'dual energy computed tomography'/syn OR ('x-ray computed tomography'/syn AND 'dual') OR ('x-ray computed tomography'/syn AND 'dual energy') OR 'dual energy computer assisted tomography'/syn  AND  'posterior cruciate ligament'/syn OR 'posterior cruciate ligament injury'/syn OR 'posterior cruciate ligament rupture'/syn OR 'posterior cruciate ligament tear'/syn OR 'anterior cruciate ligament'/syn OR 'anterior cruciate ligament rupture'/syn OR 'anterior cruciate ligament injury'/syn OR 'anterior cruciate ligament tear'/syn OR 'patellar ligament'/syn OR 'knee ligament'/syn OR 'knee medial collateral ligament'/syn OR 'lateral collateral ligament'/syn OR 'knee cruciate ligament'/syn | 56 |


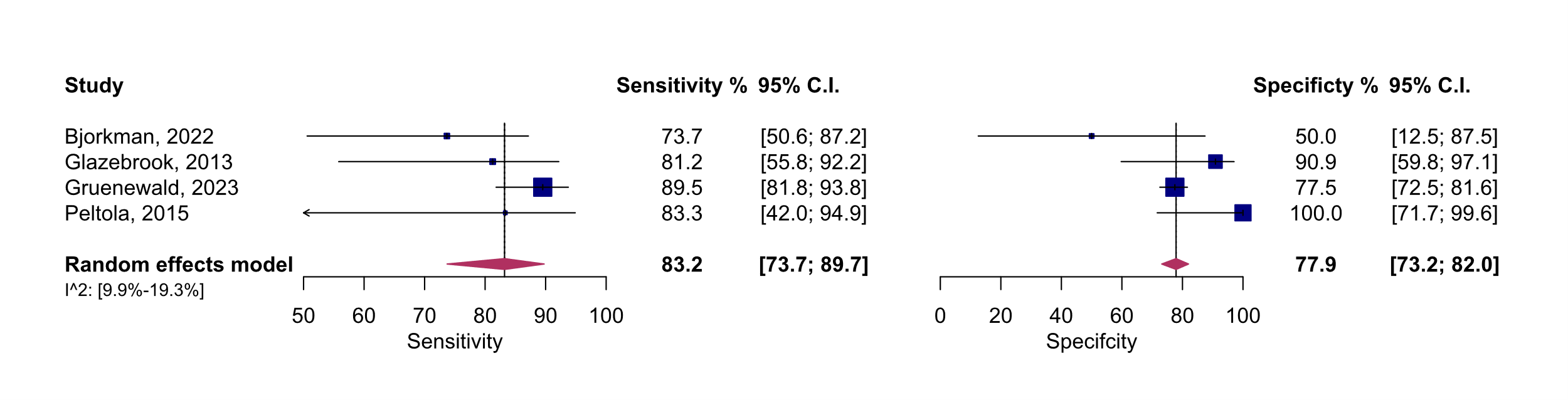


**Supplementary Fig. 1** Forest plot and summary statistics of diagnostic test accuracy (DTA) meta-analysis, after excluding one outlier study. CI. Confidence interval. EP


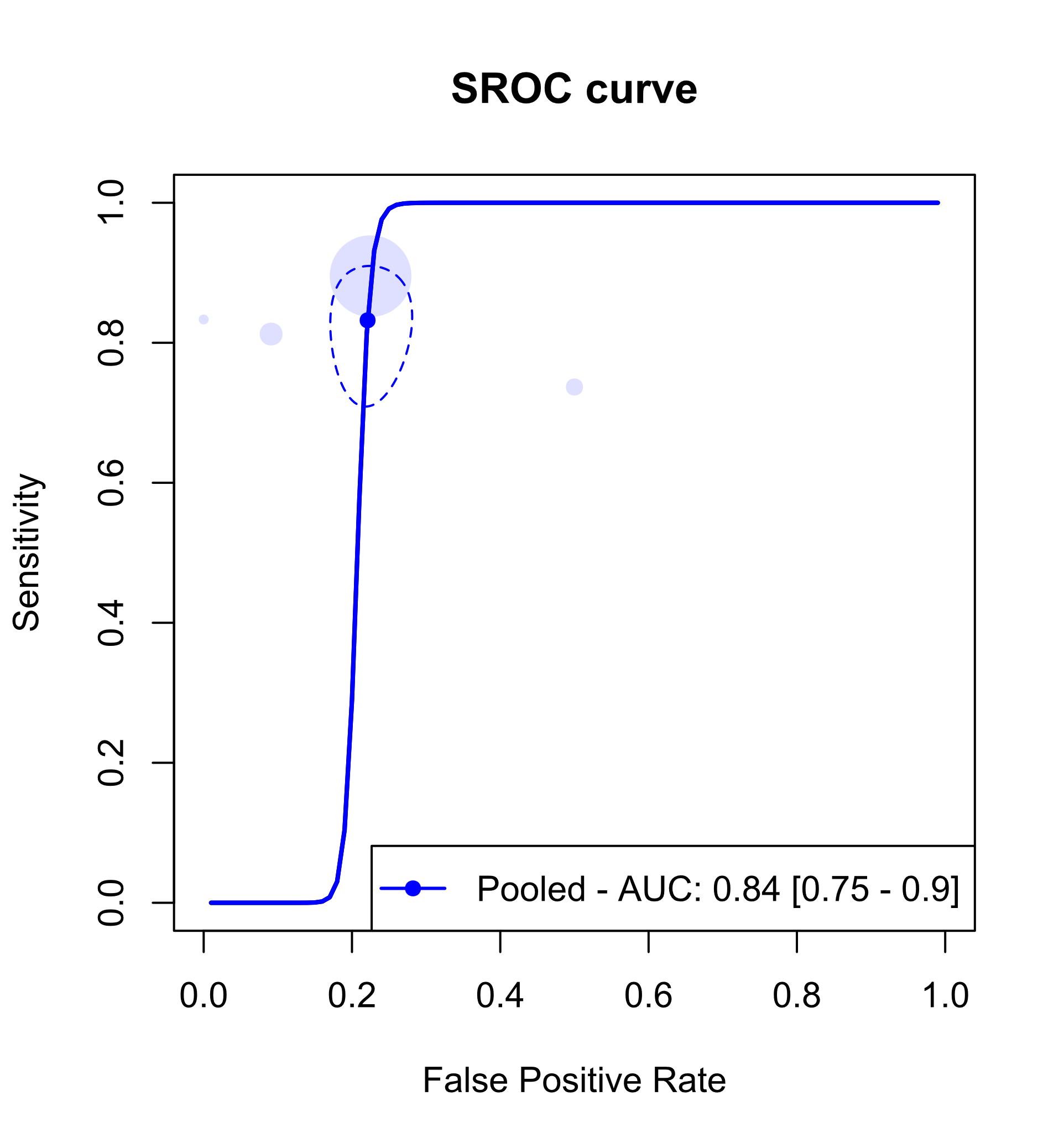


**Supplementary Fig. 2** Summary receiver operating characteristic curve (SROC) of diagnostic test accuracy (DTA) meta-analysis, after excluding one outlier study. AUC. Area under the curve. SROC. Summary receiver operating characteristic.


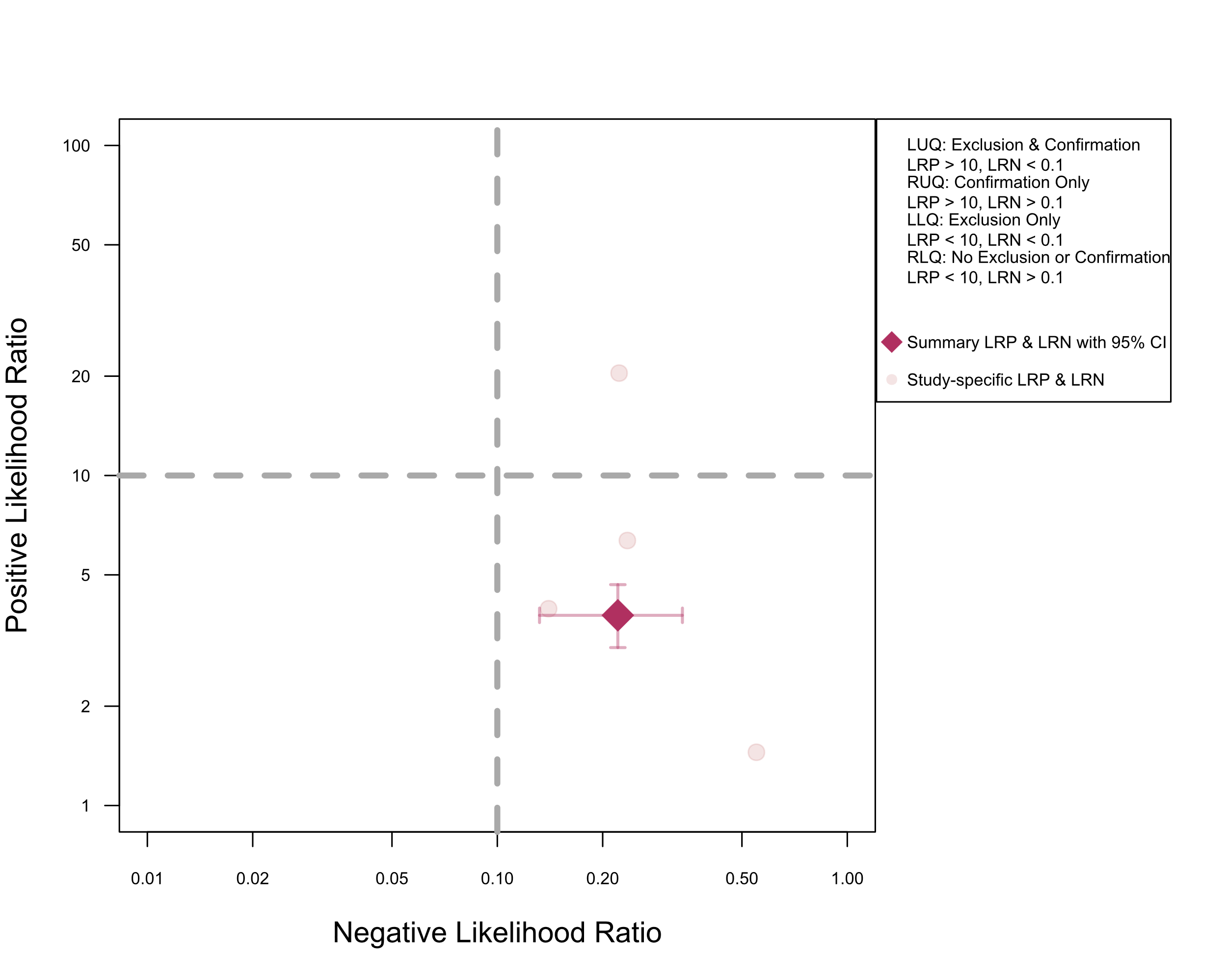


**Supplementary Fig. 3** Likelihood ratio scattergram after excluding one outlier study, indicating that the performance level is suboptimal for both exclusion and confirmation purposes. LLQ. Left lower quadrant. LRN. Likelihood ratio, negative. LRP. Likelihood ratio, positive. LUQ. Left upper quadrant. RLQ. Right lower quadrant. RUQ. Right upper quadrant.


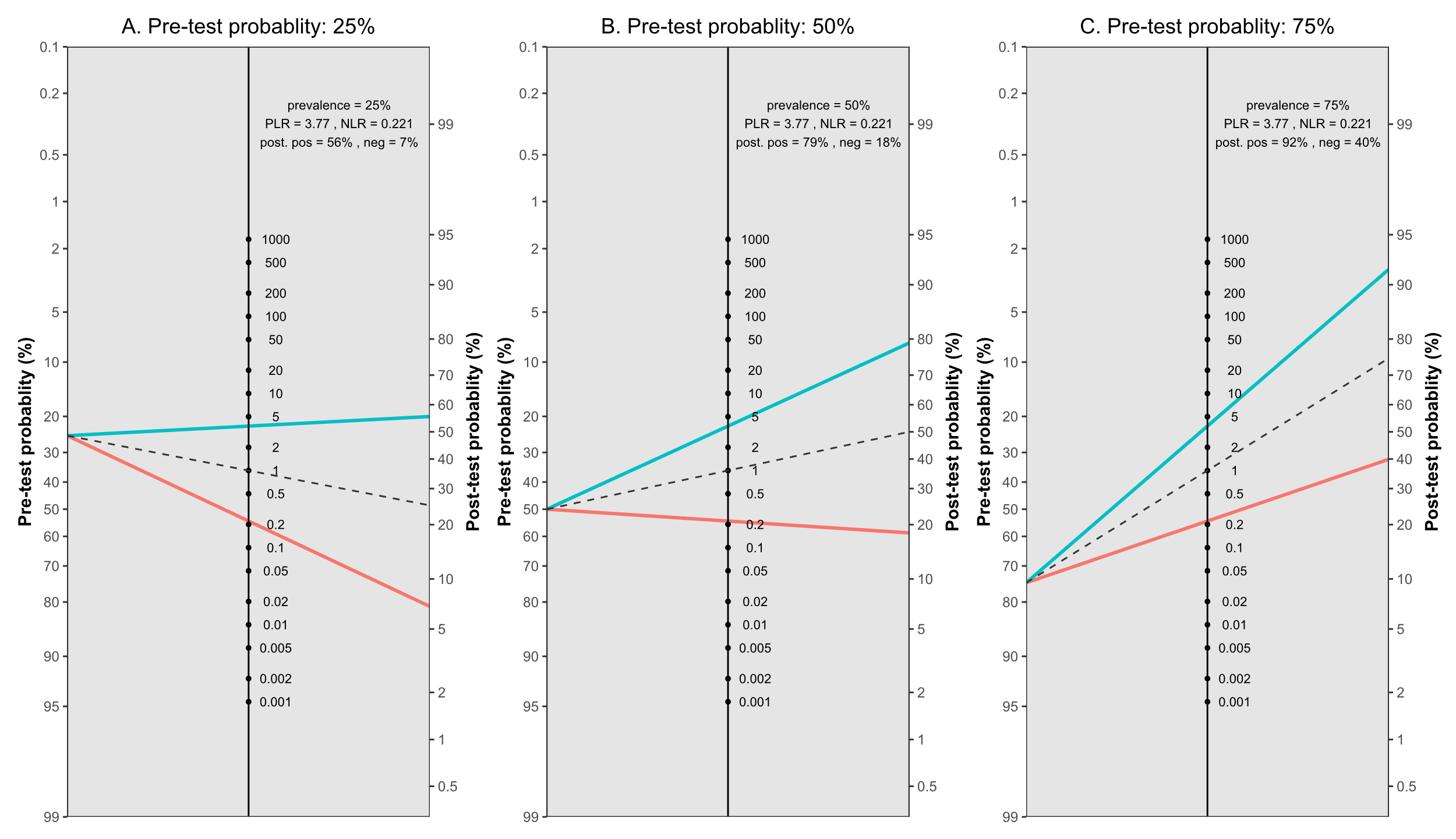


**Supplementary Fig. 4** Fagan plot analysis utilizing summary positive and negative likelihood ratio results from the meta-analysis after excluding one outlier study, considering hypothetical pre-test probabilities of 25%, 50%, and 75%. PLR. Positive likelihood ratio. NLR. Negative likelihood ratio. Neg. Negative. Pos. Positive


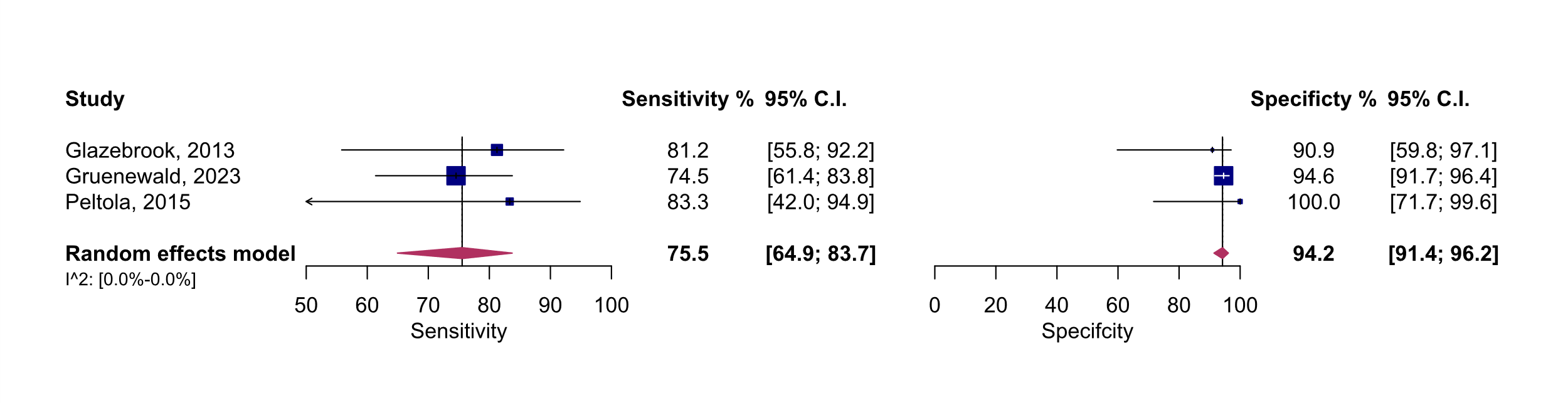


**Supplementary Fig. 5** Forest plot and summary statistics of diagnostic test accuracy (DTA) meta-analysis of studies including cases with complete rupture of anterior cruciate ligament, after excluding one outlier study. CI. Confidence interval


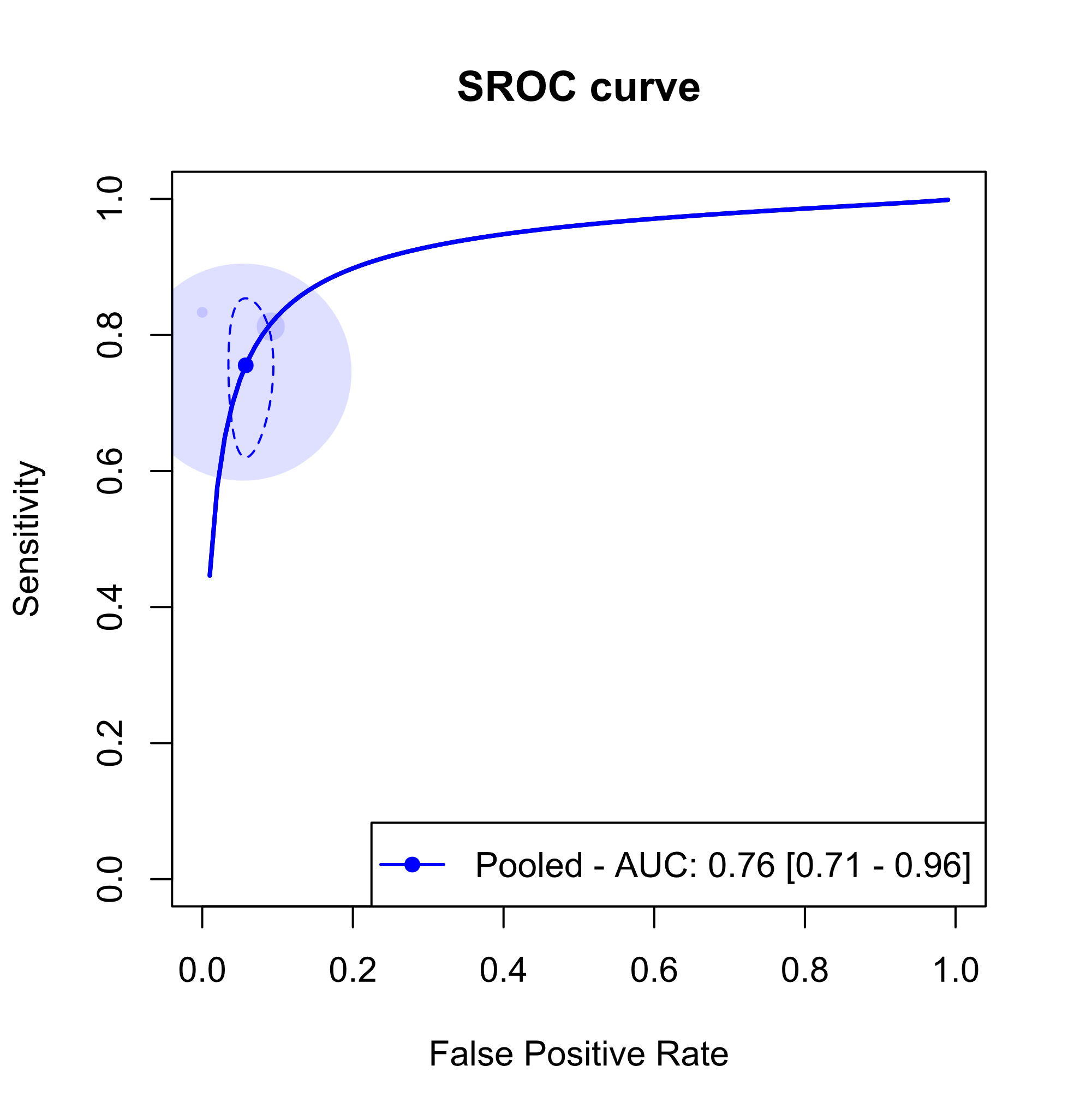


**Supplementary Fig. 6** Summary receiver operating characteristic curve (SROC) of diagnostic test accuracy (DTA) meta-analysis of studies including cases with complete rupture of anterior cruciate ligament, after excluding one outlier study. AUC. Area under the curve. SROC. Summary receiver operating characteristic.


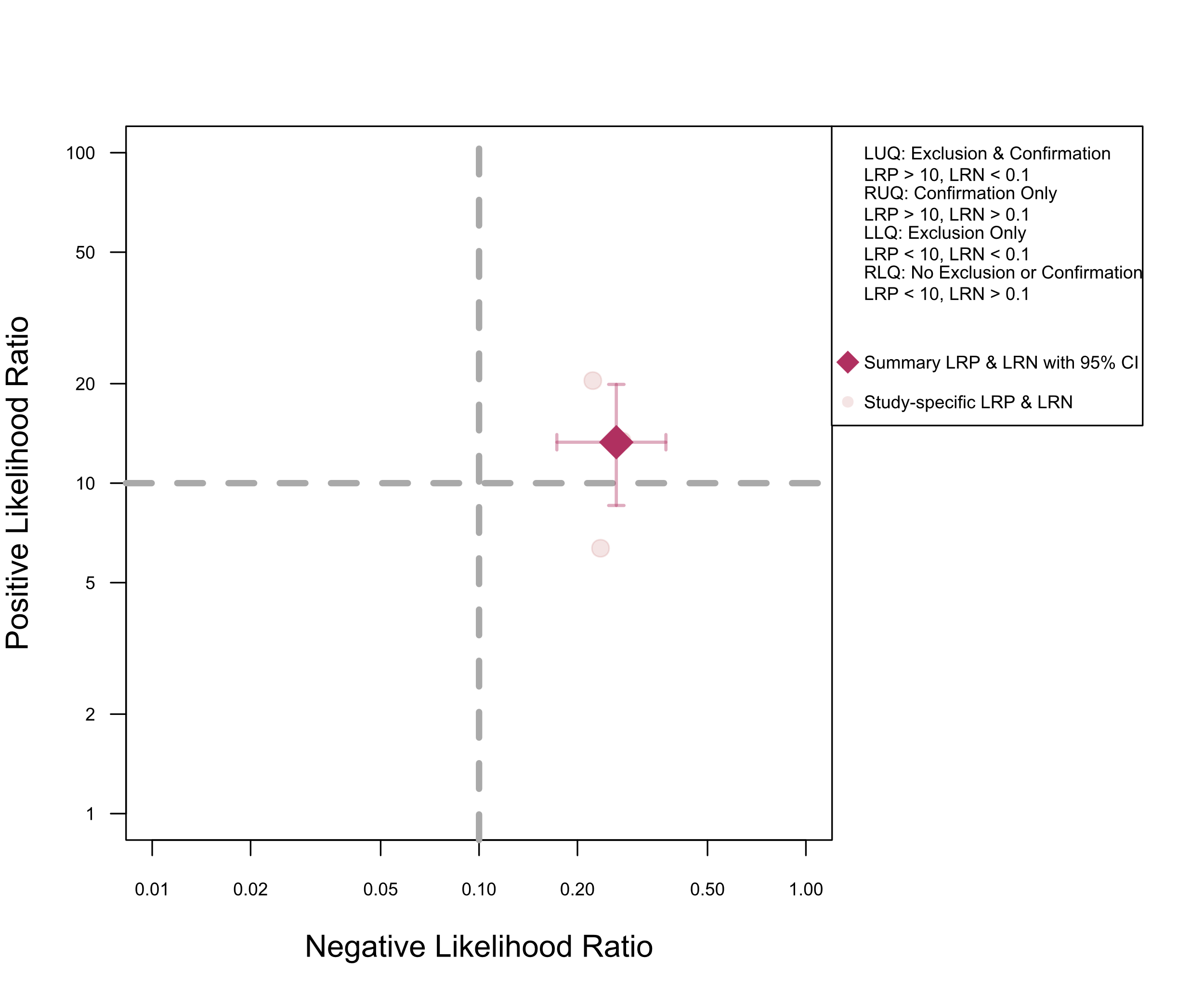


**Supplementary Fig. 7** Likelihood ratio scattergram of studies including cases with complete rupture of anterior cruciate ligament after excluding one outlier study, indicating that the performance level is optimal for only confirmation purposes. LLQ. Left lower quadrant. LRN. Likelihood ratio, negative. LRP. Likelihood ratio, positive. LUQ. Left upper quadrant. RLQ. Right lower quadrant. RUQ. Right upper quadrant


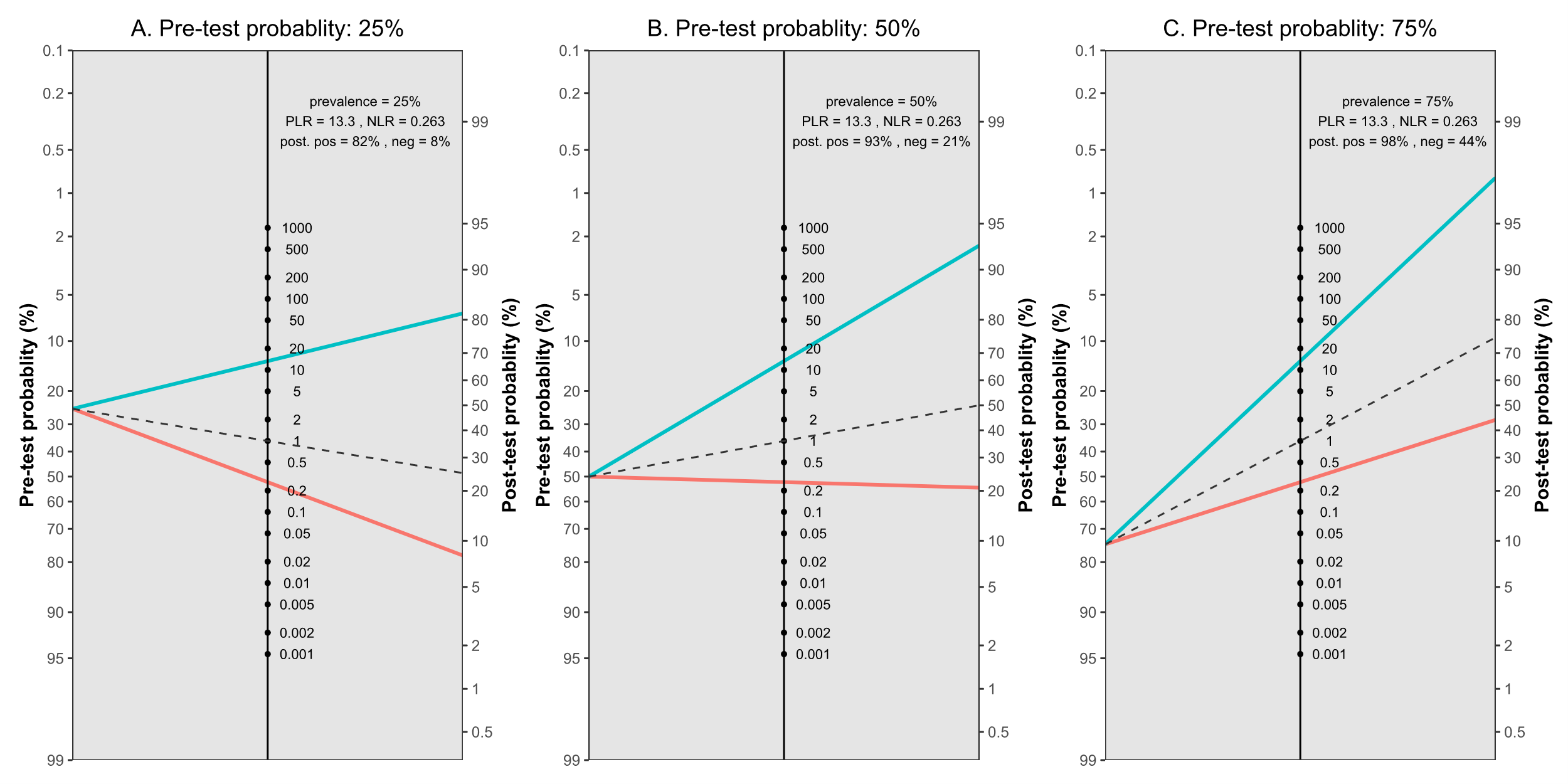


**Supplementary Fig. 8** Fagan plot analysis utilizing summary positive and negative likelihood ratio results from the meta-analysis of studies including cases with complete rupture of anterior cruciate ligament after excluding one outlier study, considering hypothetical pre-test probabilities of 25%, 50%, and 75%. PLR. Positive likelihood ratio. NLR. Negative likelihood ratio. Neg. Negative. Pos. Positive


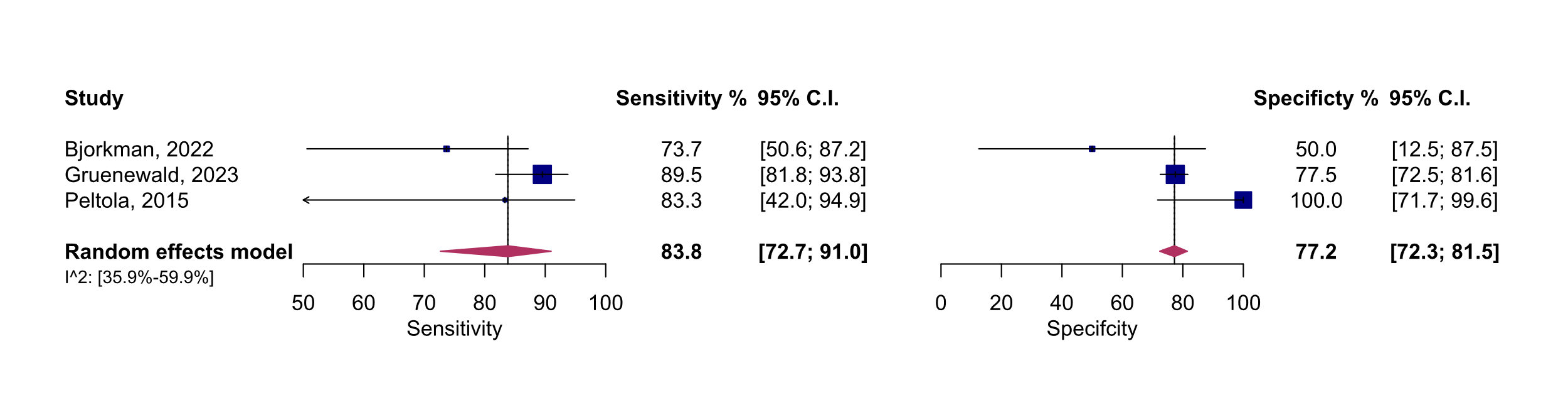


**Supplementary Fig. 9** Forest plot and summary statistics of diagnostic test accuracy (DTA) meta-analysis of studies on anterior cruciate ligament rupture in acute/subacute setting, after excluding one outlier study. CI. Confidence interval


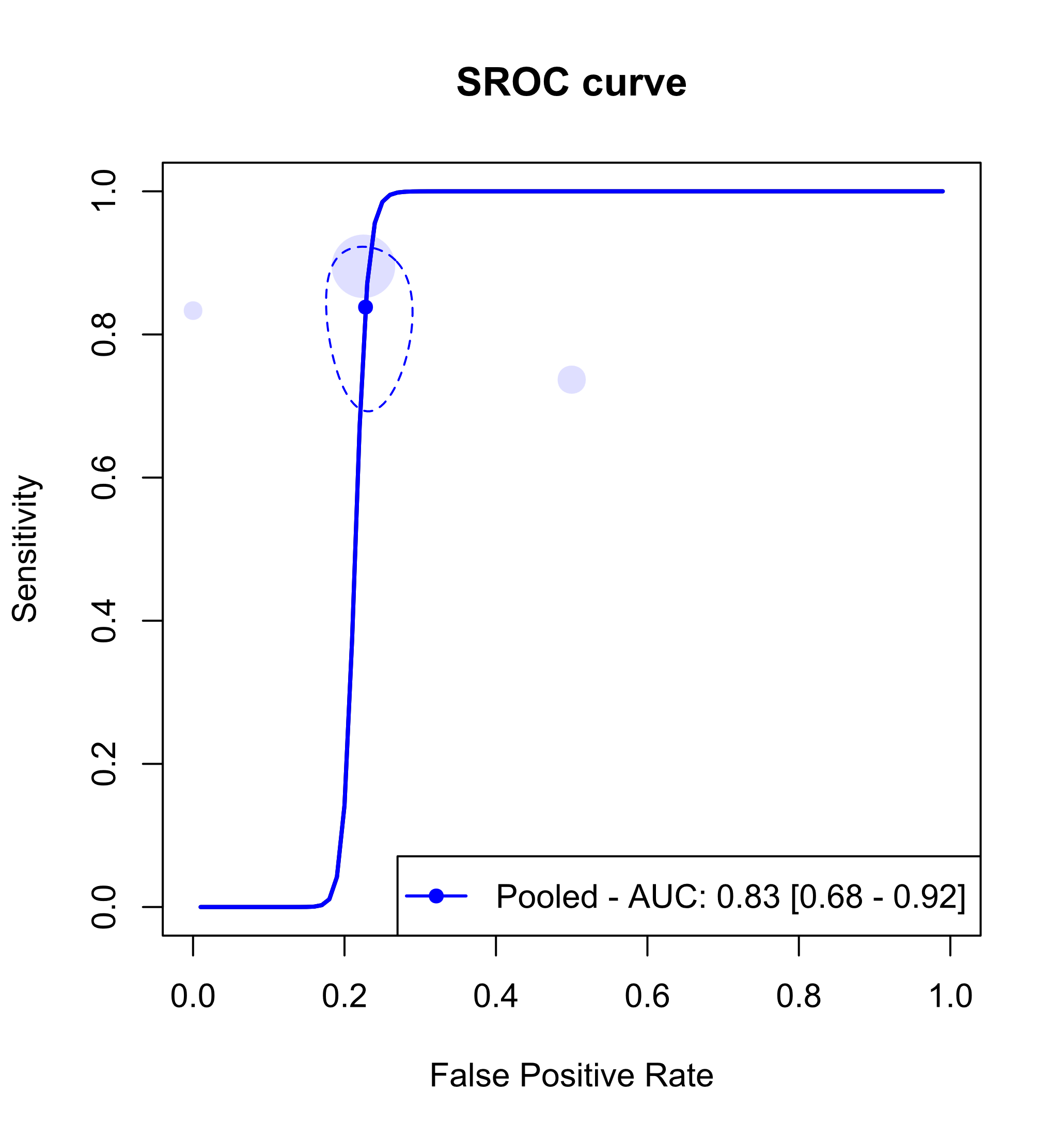


**Supplementary Fig. 10** Summary receiver operating characteristic curve (SROC) of diagnostic test accuracy (DTA) meta-analysis of studies on anterior cruciate ligament rupture in acute/subacute setting, after excluding one outlier study. AUC. Area under the curve. SROC. Summary receiver operating characteristic.

**
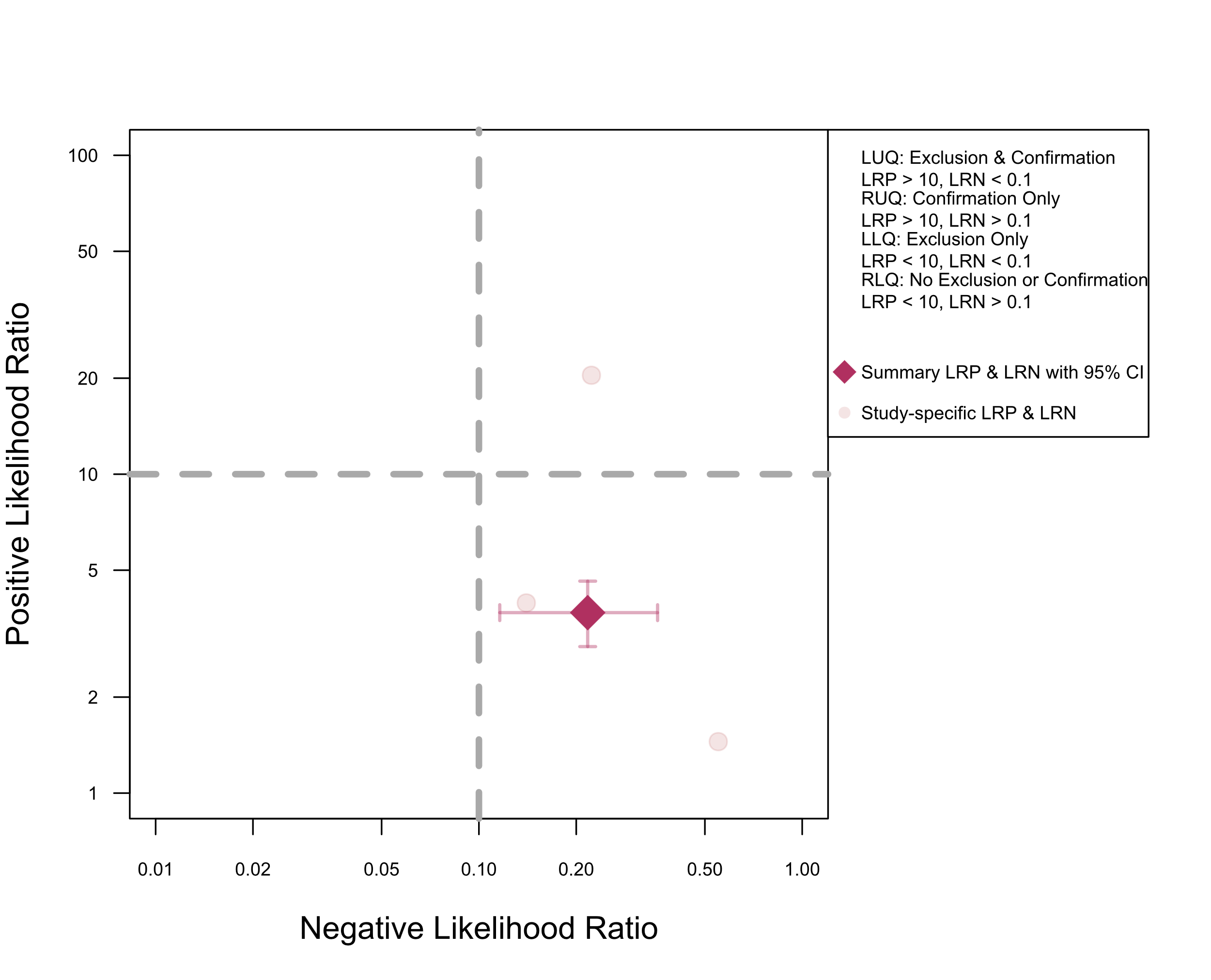
Supplementary Fig. 11** Likelihood ratio scattergram of studies on anterior cruciate ligament rupture in acute/subacute setting after excluding the outlier study, indicating that the performance is suboptimal for both exclusion and confirmation purposes. LLQ. Left lower quadrant. LRN. Likelihood ratio, negative. LRP. Likelihood ratio, positive. LUQ. Left upper quadrant. RLQ. Right lower quadrant. RUQ. Right upper quadrant


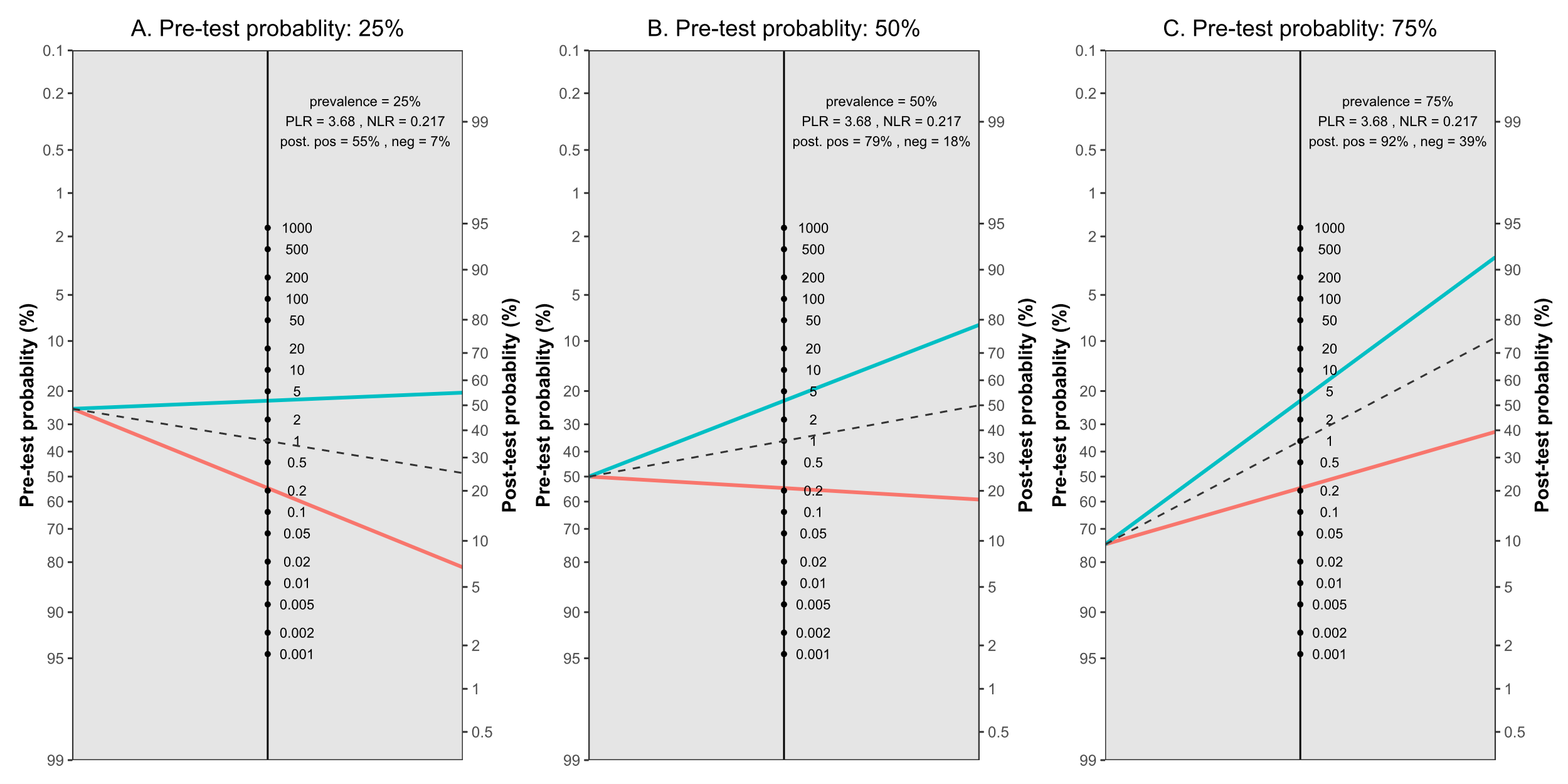


**Supplementary Fig. 12** Fagan plot analysis utilizing summary positive and negative likelihood ratio results from the meta-analysis of studies on anterior cruciate ligament rupture in acute/subacute setting after excluding one outlier study, considering hypothetical pre-test probabilities of 25%, 50%, and 75%. PLR. Positive likelihood ratio. NLR. Negative likelihood ratio. Neg. Negative. Pos. Positive
